# Coevolution of norm psychology and cooperation through exapted conformity

**DOI:** 10.1017/ehs.2024.37

**Published:** 2024-10-24

**Authors:** Yuta Kido, Masanori Takezawa

**Affiliations:** 1 Graduate School of Humanities and Human Sciences, Hokkaido University, Sapporo, Japan; 2 Japan Society for the Promotion of Science, Tokyo, Japan; 3 Center for Experimental Research in Social Sciences, Hokkaido University, Sapporo, Japan; 4 Center for Human Nature, Artificial Intelligence and Neuroscience, Hokkaido University, Sapporo, Japan

**Keywords:** Evolution, cooperation, social norm, norm psychology, cultural group selection

## Abstract

People willingly follow norms and values, often incurring material costs. This behaviour supposedly stems from evolved norm psychology, contributing to large-scale cooperation among humans. It has been argued that cooperation is influenced by two types of norms: injunctive and descriptive. This study theoretically explores the socialisation of humans under these norms. Our agent-based model simulates scenarios where diverse agents with heterogeneous norm psychologies engage in collective action to maximise their utility functions that capture three motives: gaining material payoff, following injunctive and descriptive norms. Multilevel selective pressure drives the evolution of norm psychology that affects the utility function. Further, we develop a model with exapted conformity, assuming selective advantage for descriptive norm psychology. We show that norm psychology can evolve via cultural group selection. We then identify two normative conditions that favour the evolution of norm psychology, and therefore cooperation: injunctive norms promoting punitive behaviour and descriptive norms. Furthermore, we delineate different characteristics of cooperative societies under these two conditions and explore the potential for a macro transition between them. Together, our results validate the emergence of large-scale cooperative societies through social norms and suggest complementary roles that conformity and punishment play in human prosociality.

## Graphical abstract

**Figure:**
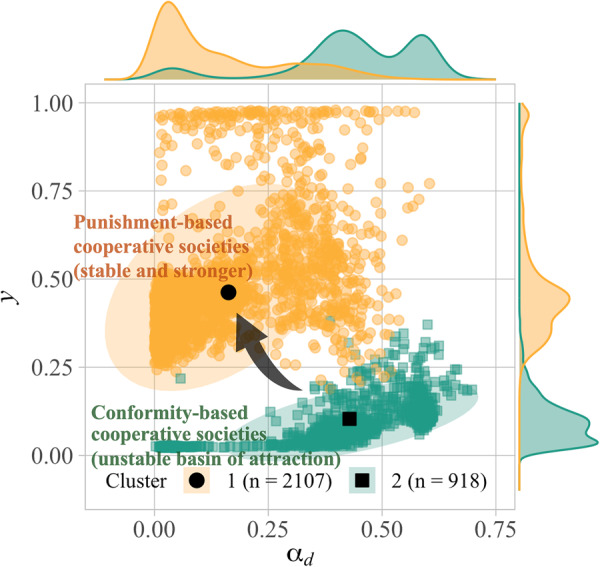

**Social media summary:** Norm-psychology can evolve, yielding two distinct states: conformity- and punishment-based cooperative societies.

## 1. Introduction

Mainstream economic theories start from the fundamental premise that humans pursue self-interest and make decisions based on rational calculations of costs and benefits (Becker, 1976). This assumption is closely related to the logic that the intrinsic gravity of evolution works in the direction of self-interest or the pursuit of survival and reproduction (Dawkins, 1982). However, this self-interested actor model systematically differs from observations: individuals anonymously donate to charity and willing incur personal costs to benefit virtual strangers. Most importantly, humans are the only species in which we observe a large-scale cooperative society among genetically unrelated ephemeral interactants. Interestingly, there is indeed large cultural variability in such cooperation (Gächter et al., 2010; Henrich et al., 2010; Herrmann et al., 2008).

Sociologists view shared societal norms as the root of large-scale cooperation unique to humans. They maintain that social norms, called ‘the grammar of society’ (Bicchieri, 2005: ix), play a central role in human behaviour by prescribing common value systems within a society (Parsons, 1951). Parsons (1937) argued that internalised norms constitute the self, biasing individuals’ behaviours toward values instilled by norms and overpowering egocentric motivations. According to this socialisation theory, cooperation follows certain norms. Likewise, cultural differences manifest because norm-prescribed behaviours differ among societies.

We find a conflict between the economic and sociological views of humankind. In economics, individuals are assumed to be rational and self-regarding, acting to maximise their payoffs, whereas in sociology, they are assumed to be highly socialised agents who prioritise internalised norms over material benefits. From a sociological perspective, it may be possible to explain the unique cooperative behaviour observed in humans. However, this poses a new puzzle: how did humans become socialised beings, deviating from the basic behavioural principle of seeking self-interest?

In this study, we develop a model that incorporates micro-macro dynamics to address this question. At the macro level, we assume two major types of social norms: injunctive and descriptive. At the micro level, that is, at the individual decision-making level, we assume that norm psychology determines one's susceptibility to the influence of social norms. Using agent-based models with evolvable norm psychology, which allow agents to internalise social norms, we explore the possibility and mechanism of coevolution between this socialisation mechanism and large-scale cooperation, as well as the condition for its occurrence.

### 1.1. Evolution of norm psychology via cultural group selection

Despite the mystery surrounding its evolution, a large body of empirical research implies that our psychologies include a predisposition to follow norms, commonly referred to as ‘norm psychology’ (Chudek & Henrich, 2011). Children are initially observed to acquire local norms within specific contexts (O'Gorman et al., 2008; Rakoczy et al., 2008), and subsequently experience activation of their brain's reward circuits when they comply with local norms (de Quervain et al., 2004; Rilling et al., 2004). This indicates that the evolved psychological mechanism allows norm compliance to be viewed as a goal rather than a burden. Accordingly, in modelling, some theorists incorporate norm psychology into the utility function (i.e. ‘norm-utility models’; see Akçay & van Cleve, 2021; Gavrilets & Richerson, 2017; Gavrilets et al., 2024; Gintis, 2014). Following their lead, our model considers the tradeoff between material utility and social preference derived from norm compliance, with the weight individuals assigned to social preferences varying depending on their norm psychologies.

To explore the evolutionary origin of norm psychology, which could pave the way for a cooperative society, our model was built on the framework of gene–culture coevolutionary accounts (Boyd & Richerson, 1985; Cavalli-Sforza & Feldman, 1981). Some authors explain the evolution of cooperation focusing on cultural processes that homogenise behaviours within groups, followed by selection among groups with large variations (referred to as ‘cultural group selection’ theory; see Boyd & Richerson, 1985; Henrich, 2004; Smith, 2020). Given high levels of migration, genetic group variations are difficult to sustain; however, cultural learning may allow for homogeneous groups and large cultural variations. If humans with altruistic genetic traits form a cooperative group, group-level selective pressure may outweigh the maladaptive nature of altruism at the individual level. In this framework, Chudek and Henrich (2011) argued that the evolution of norm psychology, which makes us socialise even under altruistic norms, was a crucial step on the path to large-scale cooperation. Following their lead, we develop a coevolutionary model that combines both cultural and genetic evolutionary processes to explain the evolution of cooperation.

### 1.2. Classification of social norms

Extensive research has been conducted on social norms and cooperation. Here, we focus on specific types of social norms, injunctive and descriptive norms, identified in previous empirical studies as important in influencing prosocial behaviour (Cialdini et al., 1990, 1991; Kallgren et al., 2000). Our models were designed to incorporate these norm features.

Injunctive norms are shared standards of behaviour that are expected in a social context. They represent exogenous rules or moral codes, transmitted to the next generation as moral values. Individuals internalising injunctive norms develop social preferences, potentially pursuing virtues benefiting groups at a cost to themselves. Studies have shown that people develop prosocial behaviour according to norms specific to their social groups (House et al., 2013, 2019, 2020; Sutter & Kocher, 2007). Theoretical studies also point to the possibility that cooperation and norm psychology, which internalise injunctive norms, have coevolved in a cooperative dilemma. According to Gavrilets and Richerson (2017), on which our model is based, injunctive norms that promote punishment are likely to be more effective in facilitating coevolutionary processes.

Descriptive norms refer to how common the behaviour is in the social setting. Unlike injunctive norms, descriptive norms depend on individual behaviour. Internalising descriptive norms leads to a preference for following the majority. In other words, it results in a form of social learning process, ‘conformity’, defined as adopting the most prevalent behaviour (Boyd & Richerson, 1985; Henrich & Boyd, 1998; Whiten et al., 2005). Substantial empirical evidence shows that descriptive norms influence cooperation (‘conditional cooperation’; e.g. Fischbacher et al., 2001) and punitive behaviour (‘conditional punishment’; Hertz, 2021). However, descriptive norms can also perpetuate detrimental or antisocial behaviours (e.g. smoking, littering, and delinquency) within a group (Schultz et al., 2007). No consistent conclusions have been drawn from theoretical studies on whether or how conformity has coevolved with cooperation (Denton et al., 2020; Efferson et al., 2016; Molleman et al., 2013; Peña et al., 2009; Romano & Balliet, 2017). Theoretical studies have shown that conformity works in tandem with punishment to promote cooperation (Andresguzman et al., 2007; Henrich & Boyd, 2001). However, our models markedly differ from previous models in assuming that a conformist learning strategy is culturally acquired depending on local environments and genetic traits, or norm psychologies. Overall, conformity is at work in real cooperative dilemmas; however, its evolutionary potential in such an environment remains uncertain.

#### Exaptation of conformity

Conformity (i.e. norm psychology of descriptive norms) was presumably selected to allow us to develop adaptive behaviours beyond cooperation. Mathematical models reveal that conformity is evolutionarily favoured under a wide range of conditions because descriptive norms serve efficiency and accuracy functions, especially in spatially and temporally variable environments (Henrich & Boyd, 1998). Evidence from various species (2-year-old children, Haun et al., 2014; primates, van de Waal et al., 2013; birds, Aplin et al., 2015) supports the idea that conformity can be regarded as a primitive capacity in our psychological mechanism. Hence, it is reasonable to assume that humans were equipped with norm psychology to internalise descriptive norms even before they faced the problem of cooperation. Therefore, just as bird feathers evolved to regulate body temperature and later adapted for flight, conformity probably exapted, evolving in other domains, and then serving one another in the domain of cooperation (Gould & Vrba, 1982). By exapted conformity, we mean a conformity that has evolved to some extent in other domains and has been brought into the domain of cooperation.

Although a large body of literature suggest evolved norm psychologies for different types of social norms underlying humans’ unique prosociality, no gene–culture coevolutionary model has addressed the questions how, why and under which conditions they evolve in interaction with each other. Here, we begin by describing our models that consider both cultural and genetic process. We built the models with the following aims, hoping to contribute to the debate about whether humans can evolve from egocentric to social agents. The first aim is to understand the nature of injunctive norms that facilitate norm psychology to coevolve with cooperation, extending the model devised by Gavrilets and Richerson (2017). Second, we explore whether norm psychology for descriptive norms (i.e. conformity) is adaptive in cooperation domains and impactful on the coevolutionary process. Third, we explore the coevolutionary scenario under the assumption of exapted conformity.

## 2. Models

We extend the agent-based model (Gavrilets & Richerson, 2017) to simulate gene–culture coevolutionary process in which individuals with heterogeneous norm psychologies engage in collective actions under the influence of two types of social norms. The major parameters in our model are shown in Table 1. This model allows us to explore the possibility that genetic evolution of norm psychology leads to cultural evolution of large-scale cooperation through socialisation of social norms. We assumed a large population of asexual individuals across groups (*G*), each consisting of 16 members (*n*). Throughout their lifetimes, group members have opportunities to participate in collective actions for over 40 rounds. The payoff structure of collective actions belongs to a general class of social dilemmas with conflicting interests between individuals and groups (see the supporting information (SI), Text S1.3 for the formulation of the payoff structure, and the SI, Figure S1-1 for the payoff function). Below, we begin by describing the cultural process that involves collective actions and social norms. We then explain the relationship between norm psychology and utility function, which drives an individual's ontogenetic plasticity. Finally, we describe the genetic evolution that optimises fitness.

### 2.1. Collective actions and social norms

We assume that agents decide whether to participate in two forms of prosocial behaviour in collective action: cooperation (denoted by variable *x*) and punishment (denoted by variable *y*). Both are binary strategies that incur a cost for the actor. The payoff *π*_*CA*_, which represents the benefit accruing from the collective action depending on the number of cooperators in the group, is distributed among all group members. Punishers harm all defectors in the group. Then, depending on each agent's strategy (*x*, *y*), strategic costs were subtracted from *π*_*CA*_, resulting in the material payoff *π*(*x*, *y*) for the individual in each round (see the SI, Text S1.3 for detailed settings regarding the strategy and material payoffs).

Following Gavrilets and Richerson (2017), we assume that these prosocial behaviours are encouraged by the injunctive norm, which is characterised by two non-negative parameters: the normative value of cooperation (denoted by variable *v*_*x*_) and the normative value of punishment (denoted by variable *v*_*y*_). We consider the injunctive norm to be exogenously given and constant throughout all generations, but the dynamics of how it is learned and adopted are endogenous to genetic and cultural evolution. This reflects a common situation across human history, in which behaviour is labelled as good or bad but enforcement is not centrally implemented. Furthermore, our models incorporated descriptive norms as typical behaviours within groups. Descriptive norms themselves change dynamically over a lifetime in the following manner: the frequency of other group members’ behaviours (including antisocial ones) in the *t*_*th*_ round determines the content and strength of the descriptive norm in the *t* + 1_*th*_ round.

### 2.2. Norm psychology and strategy revision

We consider agents that update their strategies with a probability of 0.25 every round throughout their lifetime based on the myopic optimisation algorithm (Sandholm, 2010), which means that individuals choose the optimal strategy based on the others’ ones in the previous round, producing the best response dynamics. Individuals revise a combination of strategies (*x*, *y*) to maximise the following utility function, considering both material payoffs and social norms:1
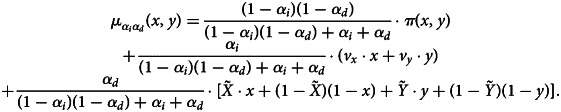


We assume that the influence of social norms is determined by the norm psychology parameter (denoted by *α* ∈ [0.0, 1.0]). We clearly distinguish between norm psychology of injunctive norm (denoted by *α*_*i*_) and descriptive norm (denoted by *α*_*d*_). These genetic traits (*α*_*i*_ and *α*_*d*_) evolve biologically, allowing for heterogeneously socialised agents and therefore changes in the optimal strategy. The first term in Equation (1) corresponds to the preference for material payoff *π*(*x*, *y*) that an agent obtains in each round. Low values of *α*_*i*_ and *α*_*d*_ make agents payoff-oriented because agents place the weight on this preference depending on the value of (1 − *α*_*i*_)(1 − *α*_*d*_). Agents with high value of *α*_*i*_ have a greater weight in the second term. In other words, they obtain higher utility by following the injunctive norm, whose content is characterised by the exogenous parameters *v*_*x*_ and *v*_*y*_. On the other hand, 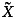 and 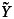 are the frequencies of each strategy among other group members, corresponding to the strength of descriptive norms. Altogether, agents with high value of *α*_*d*_ assign more weight to the third term and get higher utility from conforming to others. Note that descriptive norms can encourage selfish behaviours (i.e. *x* = 0, *y* = 0) in our models when few other group members adopt a prosocial strategy (i.e. low values of 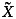 and 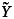), in contrast to injunctive norms, which encourage only prosocial behaviours (see the SI, Text S1.3 for the detailed settings of the strategy revision algorithm).

**Table 1.** Parameters

Symbol	Definition	Value
*G*	Number of groups	500
*n*	Group size	16
*T*	Number of generations	30,000
*x*	Behavioural trait of cooperation	Variable in {0, 1}
*y*	Behavioural trait of punishment	Variable in {0, 1}
*v*_*x*_	A constant determining the strength of injunctive norm for cooperation	{0.0, 0.1, …, 1.0}
*v*_*y*_	A constant determining the strength of injunctive norm for punishment	{0.0, 0.1, …, 1.0}
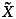	Frequency of cooperators among other group members	Variable in [0.0, 1.0]
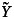	Frequency of punishers among other group members	Variable in [0.0, 1.0]
*α*_*i*_	Genetic trait of injunctive norm-psychology	Evolvable in [0.0, 1.0]
*α*_*d*_	Genetic trait of descriptive norm-psychology	Evolvable in [0.0, 1.0]

### 2.3. Multilevel selection pressure on norm psychology

Each individual was characterised by genetic traits, denoted by *α*_*i*_ and *α*_*d*_. The initial values are taken from the uniform distribution of (*α*_*i*_, *α*_*d*_) ∈ [0, 0.05]^2^ with the exception of the exaptation of conformity assumed in the model (higher initial distribution of *α*_*d*_; Table 2, Model 3). This means that the population starts with self-interested agents with poor socialisation abilities. The evolution of norm psychology is governed by natural selection. Depending on their success in life, multilevel selection drives the evolution of norm psychology. Selection follows a two-level Wright–Fisher process; thus, generations are discrete and nonoverlapping. First, the population in the previous generation is subject to group-level selection, captured by the replication of group *j* with a probability proportional to the group fitness 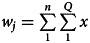, given by the cumulative number of cooperators across 40 rounds (*Q*) among 16 group members (*n*). This means that the more benefits a group accumulates, the more likely it is to survive and replicate. Second, the population that survived group-level selection is subject to individual-level selection, captured by the reproduction of individual *i* with probability proportional to 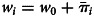, given by the addition of baseline fitness *w*_0_ and mean of payoffs 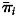.

**Table 2.** Model assumptions

	Model 1	Model 2	Model 3
Assumed norm psychology	*α*_*i*_	*α*_*i*_ and *α*_*d*_	*α*_*i*_ and *α*_*d*_
Initial distribution of *α*_*i*_	~*U*(0.00, 0.05)	~*U*(0.00, 0.05)	~*U*(0.00, 0.05)
Initial distribution of *α*_*d*_		~*U*(0.00, 0.05)	~*N*(0.30, 0.25^2^)

*Note:* Model 1 is essentially the same as Gavrilets and Richerson's (2017) model. Models 2 and 3 are built upon Model 1 with the addition of the non-exapted and exapted forms, respectively, of *α*_*d*_.

Under self-interested rationality, individuals would be better off pursuing a material payoff than internalising social norms. Consequently, natural selection at the individual level always decreases reliance on norm psychology. Importantly, however, all agents inhabit a shared environment, which can be influenced by the behaviour of others. If there are many agents around who enforce norms through punishment, defection (*x* = 0) is no longer the optimal strategy; if there are many cooperators around (i.e. large 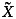), agents with high *α*_*d*_ potentially obtain the highest utility from cooperation (*x* = 1). In other words, through this coexistence, norm psychology can make groups cooperative, resulting in more success than uncooperative groups, so that norm psychology can be favoured at the group level, although not always (see the SI, Text S1.3 for detailed settings of the multilevel selection algorithm). Finally, half of the group members were randomly selected from each group and migrated to other groups.

## 3. Results

We consider three models with different settings for social norms. We begin by assuming only injunctive norms and norm psychology *α*_*i*_, but go on to investigate the impact of descriptive norms and *α*_*d*_ (Table 2). More precisely, first, we replicate the simulation of the model (Gavrilets & Richerson, 2017; Table 2, Model 1) that assumes injunctive norms. However, our simulation differs from previous models in its more fine-grained manipulation of the injunctive norms. Specifically, we exogenously gave normative values for each prosocial behaviour (*x*, *y*), varying from 0 to 1 in intervals of 0.1 as (*v*_*x*_, *v*_*y*_), yielding 121 simulated combinations of injunctive norms (much more than nine combinations in Gavrilets and Richerson (2017) that have each normative value 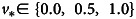). Second, we extended Model 1 by allowing for the influence of descriptive norms and the corresponding norm psychology *α*_*d*_ (Table 2, Model 2). Third, we modelled the exapted conformity by setting an initial distribution of *α*_*d*_ higher as normal distribution *N*(0.30, 0.25^2^) (Table 2, Model 3). Note that the results of Model 3 do not depend, qualitatively, on the specific shape of initial distributions (see the SI, Figure S1, for results under other assumptions about exaptation).

**Figure 1.**
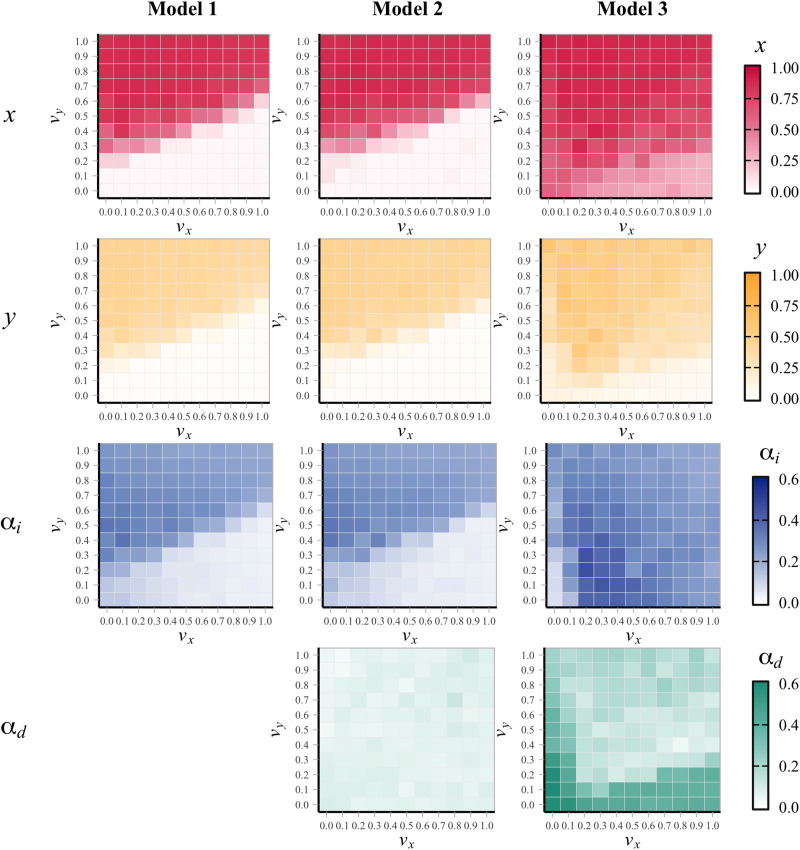
Summary results. Heatmap of *x* (cooperation), *y* (punishment), *α*_*i*_ (injunctive norm psychology) and *α*_*d*_ (descriptive norm psychology) for different normative values, *v*_*x*_ (injunctive norm for cooperation) and *v*_*y*_ (injunctive norm for punishment) and three models with different assumptions (Table 2). Shown are averages based on 25 runs for each parameter combination. As for results under other assumptions about exaptation, see the SI, Figure S1.

In the analysis, we consider both the steady-state values, approximated by the values in the last generation, and the temporal dynamics. As for behavioural data (i.e. *x*, *y*), we report the frequency in the last round. All simulations were routinely run for 30,000 generations to ensure that the genetic traits and resulting behavioural traits reached a steady state as much as possible. In Figure 1, the summary results are illustrated based on the mean value of the last generation for 25 simulation runs.

### 3.1. Results of Models 1 and 2

For the results of the Model 1 (Figure 1, left column), the third row of the heatmap shows that genetic trait *α*_*i*_ evolves to some extent (0.2 < *α*_*i*_ < 0.4) in the top-left region of the parameter space (*v*_*x*_, *v*_*y*_). High cooperation rates (0.8 < *x*) and intermediate levels of punishment (0.3 < *y* < 0.5) were observed in the same parameter regions. These observations on the normative conditions for coevolution are broadly consistent with previous findings that cooperation readily evolves under injunctive norms that encourage punishment; in contrast, promoting cooperation is not effective (Gavrilets & Richerson, 2017). Furthermore, the increased precision of injunctive norms reveals a new finding: when the norm explicitly values cooperation strongly (i.e. high *v*_*x*_), *α*_*i*_ tends not to evolve and then, paradoxically, neither does cooperation. In particular, cooperation almost never emerges (mean *x* ≈ 0.03) under (*v*_*x*_, *v*_*y*_) = (1.0, 0.5). The *x*, *y* and *α*_*i*_ values do not markedly differ between Models 1 and 2, while *α*_*d*_ remains very small (Figure 1, middle column). This result suggests that *α*_*d*_ that leads to conformist learning is not favoured in the cooperation domain and, thus, does not influence other evolutionary dynamics and cultural equilibria. In summary, the evolution of cooperation requires an injunctive norm for punishment (*v*_*y*_) that is sufficiently larger than that for cooperation (*v*_*x*_) under the non-exapted conformity assumption (Models 1 and 2). In the following subsection, we examine why injunctive norms for punishment are prerequisites for coevolution.

#### Evolutionary dynamics (coevolution of *α*_*i*_ and cooperation)

**Figure 2.**
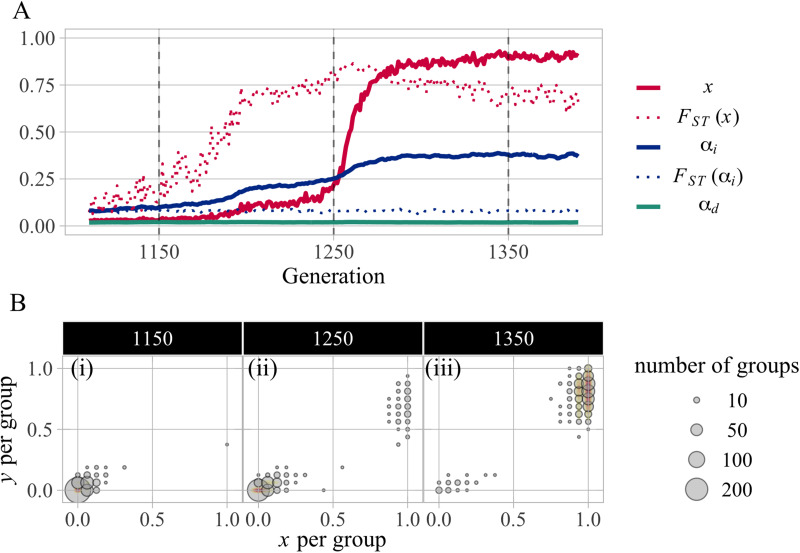
Example of evolutionary dynamics under the setting of non-exapted *α*_*d*_ (Model 2) with (*v*_*x*,_ *v*_*y*_) = (0.5, 0.5). (a) Mean of *α*_*i*_ (blue solid), genetic *F*_*ST*_ of *α*_*i*_ (blue dotted), mean of *α*_*d*_ (green solid), cooperation (red solid) and behavioural *F*_*ST*_ of cooperation (red dotted) over the specific generations for a representative simulation. (b) Rate of each behaviour among 500 groups in each generation, with the size representing the number of groups that have the same frequencies of behaviours, *x* and *y*. Here, we narrowed down 30,000 to about 300 generations, but afterwards a steady state was reached with some fluctuations (see the SI, Figure S2, for the dynamics over all generations).

Figure 2a illustrates the evolutionary dynamics typically observed through a representative run (under the setting of (*v*_*x*_, *v*_*y*_) = (0.5, 0.5), where cooperation evolved robustly). Here, we draw on the established metric *F*_*ST*_ that represents the degree of genotypic differentiation between subpopulations to measure the variation of phenotype of cooperation between groups (red dotted line in Figure 2a). The *F*_*ST*_ values range from 0 to 1, with higher values indicating greater differentiation between groups (see the SI, Text S1.3 for the detailed formulation of *F*_*ST*_). For example, when the population is polarised into all-cooperator and all-defector groups, the value of *F*_*ST*_ equals 1, whereas it equals 0 when the proportion of cooperators is uniform across all groups. We observed the following dynamics: first, the capacity to internalise the injunctive norm *α*_*i*_ evolves over time (up to the 1200th generation); second, this evolution leads to an increase in cooperative behaviour *F*_*ST*_ (1150th–1250th generations). Ultimately, the cooperation rate rises dramatically (up to the 1250th generation). Notably, the genetic *F*_*ST*_ (blue dotted line in Figure 2a) remains small while the behavioural *F*_*ST*_ is large. These observations are consistent with the evolutionary dynamics of cooperation based on cultural group selection theories (Henrich, 2004) and empirical findings (Bell et al., 2009). Furthermore, the group variations at the three time points (Figure 2b) show the significant effect of punishment on the number of cooperators in the group. In particular, if more than half of the punishers belonged to a group, most group members cooperated. Thus, our results reveal that a fraction of norm enforcers can emerge and shape a cooperative group, which ultimately drives the process in line with the predictions of cultural group selection theory.

### 3.2. Result of Model 3

Now, consider the case of exapted conformity (Table 2, Model 3). This time, suppose that simulations start with a population whose initial values of genetic trait *α*_*d*_ are randomly sampled from *N*(0.3, 0.25^2^). Comparisons of the results with Models 2 and 3 explicitly indicate that the latter is more conducive to the coevolution of norm psychology and cooperation. Under the assumption of exapted conformity, cooperation and punishment evolve at high frequencies over a much wider range of conditions than in the other two models. Even when the injunctive norm does not encourage prosocial behaviour at all (i.e. (*v*_*x*,_*v*_*y*_) = (0.0, 0.0)), intermediate level of cooperation is observed (*x* ≈ 0.60, *y* ≈ 0.12 and *α*_*d*_ ≈ 0.59). Moreover, under (*v*_*x*,_*v*_*y*_) = (1.0, 0.5), where cooperation did not evolve in Model 1 and Model 2, *x* becomes greater than .7 with *y* ≈ 0.32 and *α*_*i*_ ≈ 0.26. These results are probably due to the synergistic relationship between exapted conformity and injunctive norms. However, finding a pattern for these effects based on the average of all the simulations (Figure 1) was a challenge. Thus, we classified all simulation results for the last generation based on all parameter values *x*, *y*, *α*_*i*_, and *α*_*d*_, using clustering analysis by *k*-means method, which suggests that there are two distinct clusters.

**Figure 3.**
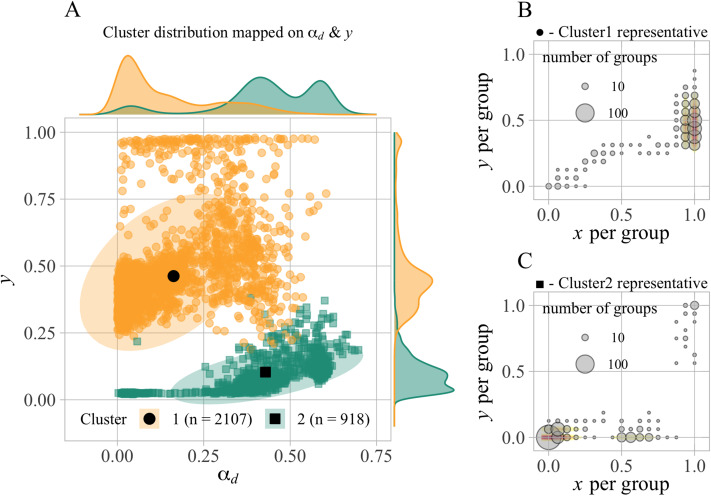
Clustering of all results under the setting of exapted *α*_*d*_ (Model 3). (a) Scatterplot of all simulation results for the last generation, with the mean value of *α*_*d*_ and the frequency of *y* on the axes, clustered by the *k*-means method. Results are plotted as yellow circles for Cluster 1 and as green squares for Cluster 2. Ellipses cover about 80% of simulations in each cluster, assuming a multivariate normal distribution. The simulations closest to the centroid of each cluster are shown in the black circle (Cluster 1) and square (Cluster 2). (b, c) Frequency of *x* and *y* per group in the representative simulation (i.e. the centroid of each cluster) with the size showing the number of groups whose frequencies of behaviours were the same. As for the analysis of optimal number of clusters and clustering results for Model 2, see the SI, Figure S8.

Figure 3 plots the mean value of *α*_*d*_ and the mean frequency of punishment *y* for each of 25 runs in each injunctive normative condition. Obviously, the two clusters are characterised by a combination of two parameters, *α*_*d*_ and *y*. In the first cluster (hereafter, Cluster 1), *α*_*d*_ evolves to a surprisingly small value (mean *α*_*d*_ ≈ 0.14) with a certain number of punishers (mean *y* ≈ 0.48) among the population; in the second cluster (hereafter, Cluster 2), exapted *α*_*d*_ remains high or evolves to a higher value (mean *α*_*d*_ ≈ 0.43) with few punishers (mean *y* ≈ 0.10). Cooperation is observed in both clusters, although its extent and mechanisms differ.

To better understand the nature of the two stable states, we identified the simulations closest to the centroid of each cluster (represented by the black points in Figure 3a), and presented the frequency of cooperators and punishers per group in the final generation (Figure 3b, c). Cluster 2, in which conformists are not driven out, is common when either the injunctive norm does not strongly promote punishment (low values of *v*_*y*_) or does not encourage cooperation (*v*_*x*_ = 0.0) (see the SI, Figure, S10b for the relationship between normative values and the cluster ratio in Model 3). In these instances, intermediate levels of cooperation with large group variation are achieved by exapted conformity alone, where low levels of punishment are observed because descriptive norms of punishment are rarely formed and maintained, owing to a net negative cost of punishment. On the other hand, in broader conditions, the population settles down into Cluster 1, characterised by higher *y* and lower *α*_*d*_. Figure 3b shows a clear trend in which cooperation is achieved with the punishers. The societies tend to achieve higher cooperation when supported by punishment than by conformity (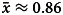 in the Cluster 1, while 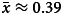 in the Cluster 2 on average). Moreover, the assumption of exapted conformity expands the basin of attractions for punishment-based cooperative societies (Cluster 1), as well as conformity-based societies (Cluster 2). Then, we explore the dynamics behind the macroscopic change that results in the evolution of cooperation over a wider range of conditions by scrutinising a simulation in the normative condition (*v*_*x*,_ *v*_*y*_) = (1.0, 0.5), in which cooperation evolves robustly only in Model 3.

#### Evolutionary dynamics (coevolution of *α*_*i*_, *α*_*d*_ and cooperation)

**Figure 4.**
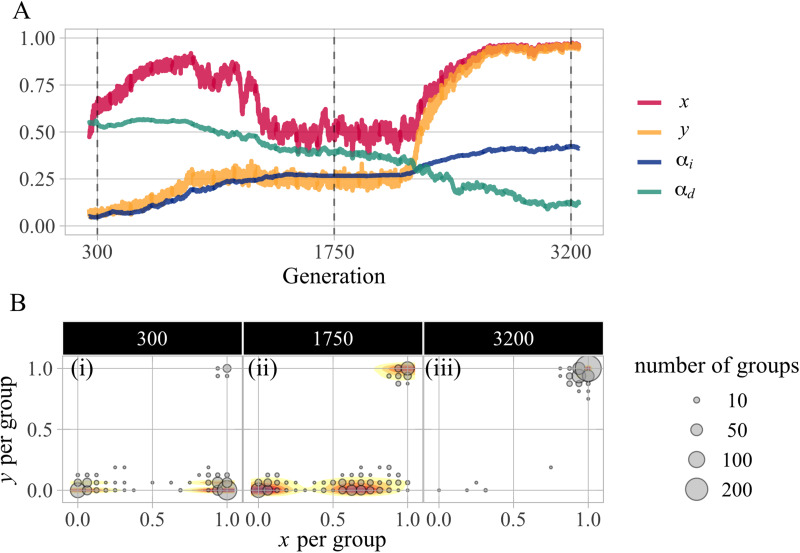
Example of evolutionary dynamics under the setting of exapted *α*_*d*_ (Model 3) with (*v*_*x*_, *v*_*y*_) = (1.0, 0.5). (a) Mean of *α*_*i*_ (blue), *α*_*d*_ (green), *x* (red), and *y* (yellow) over the specific generations for a simulation. (b) Rate of each behaviour among 500 groups in each generation, with the size representing the number of groups whose frequencies of behaviours were the same. Here, we narrowed down 30,000 to about 3000 generations. Note that thereafter the frequency of punishers decreases by about half, and the mean value of *α*_*i*_ also decreases slightly, reaching a steady state (see the SI, Figure S5, for the dynamics over all generations).

Here, through typical temporal dynamics, we show that the mechanism underlying cooperative societies shifts from conformity (descriptive norm) to punishment (injunctive norm). Figure 4 illustrates the dynamics over approximately 3000 generations in an exemplary simulation under the setting of (*v*_*x*,_ *v*_*y*_) = (1.0, 0.5). In Figure 4a, we observe a series of dynamics consisting of the following three phases: First, a cooperative state with high *α*_*d*_ and low *y* is achieved (up until the 300th generation). In this phase, the maintenance of cooperation depends on conformist learning, causing significant cultural differences in cooperation between groups, even without punishment (Figure 4b(i)). However, this state, which can be classified as a conformity-based cooperative society (Cluster 2), does not last long. Instead, a transition occurs in the social state. This is the second phase of the dynamics. During this phase, the mechanism for maintaining cooperation shifts from conformity to punishment, with a temporary decline in cooperation to approximately 50% (around the 1000th–2000th generation). This is illustrated in Fig. 2b(ii), where punishment-based cooperative groups begin to emerge and all agents adopt both prosocial strategies (i.e. *x* = *y* = 1). Over the course of time, *α*_*i*_ and prosocial behaviours increase considerably at a certain tipping point (around the 2000th generation). Finally, a cooperative society relying on high *α*_*i*_ and *y* emerges, with very low values of *α*_*d*_ (around the 2500th generation). In Figure 2b(iii), almost all the groups converge to a state with high *x*, *y*.

**Figure 5.**
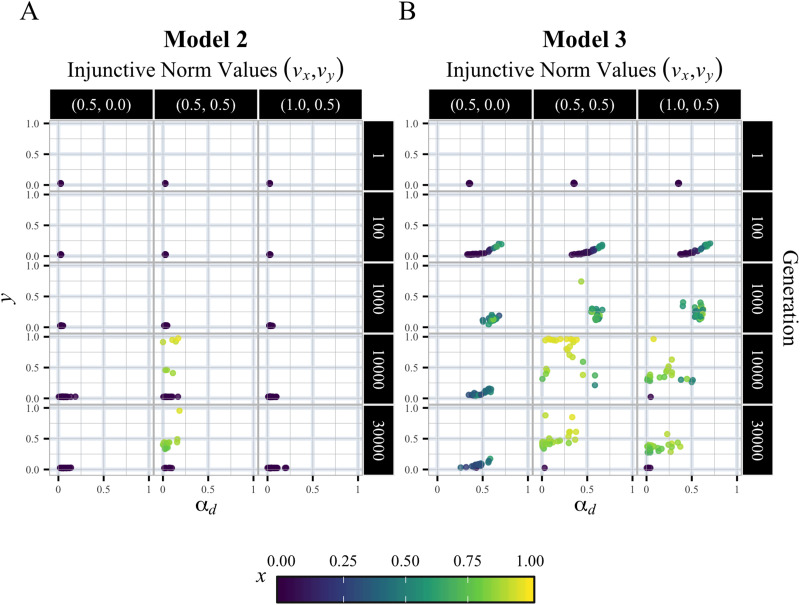
Comparison of temporal dynamics between Models 2 and 3. (a, b) Trajectory of 25 runs at 5 time points (1, 100, 1000, 10,000 and 30,000th generation) in the 2D space of *α*_*d*_ and *y*, for three combinations of injunctive norm values (*v*_*x*_, *v*_*y*_) = (0.5, 0.0), (0.5, 0.5), (1.0, 0.5) in Models 2 and 3. Colour represents cooperation rate at each time point.

Here, we examine the generality of the phase transition dynamics from conformity-based to punishment-based cooperation observed in Figure 4. Figure 5 plots the 2D state transitions of 25 runs in *α*_*d*_ and *y* space at 5 time points for three norm value (*v*_*x*_, *v*_*y*_) combinations. In Model 2, cooperation only evolved when (*v*_*x*_, *v*_*y*_) = (0.5, 0.5), all of which converge to the punishment-based state. In Model 3, cooperation also evolved when (*v*_*x*_, *v*_*y*_) = (0.5, 0.5), but pathways to cooperative states markedly differ. In early generations all runs are in the state of cooperation by conformity, and over generations they transition to cooperation by punishment in the upper left of the pane. Moreover, while no runs showed the evolution of cooperation in Model 2 for (*v*_*x*_, *v*_*y*_) = (1.0, 0.5), most of the runs in Model 3 exhibited the similar trajectory leading to cooperation.

**Figure 6.**
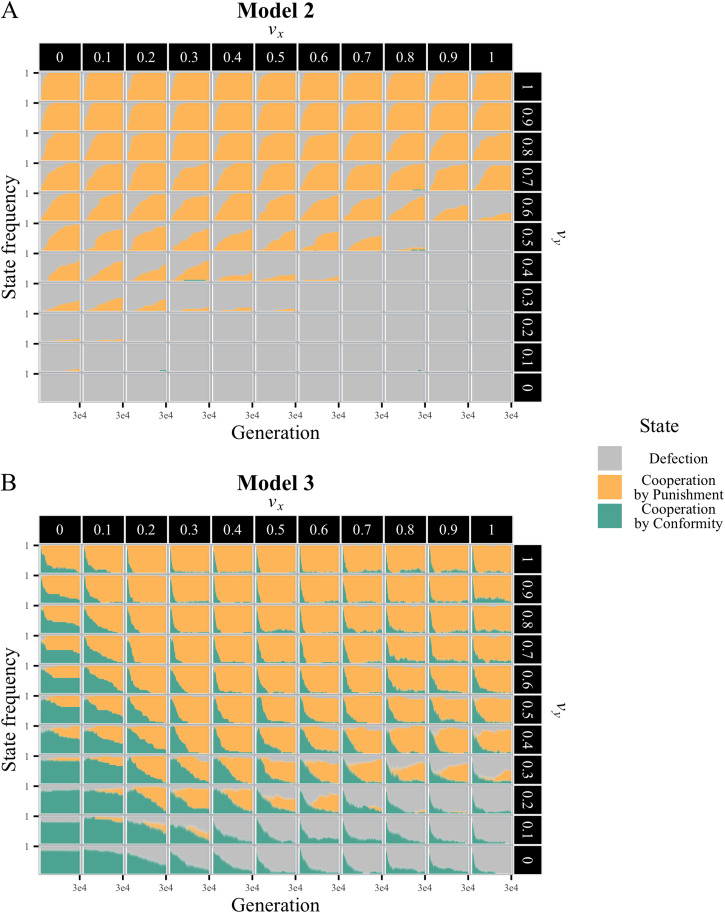
Comparison of state transition dynamics between Models 2 and 3. (a, b) Time series of frequencies of the following three states across all injunctive norm value combinations (*v*_*x*_, *v*_*y*_). ‘Defection’ (grey) is defined as *x* < 0.5, ‘cooperation by punishment’ (yellow) as *x* ≥ 0.5 *and y* ≥ *α*_*d*_, and ‘cooperation by conformity’ (green) as *x* ≥ 0.5, *α*_*d*_  >  *y*.

We further categorised run states into ‘defection’ (*x* < 0.5), ‘cooperation by punishment’ (*x* ≥ 0.5 *and y* ≥ *α*_*d*_), and ‘cooperation by conformity’ (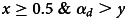), and illustrated their frequency changes over full generations (Figure 6). In Model 2, punishment-based cooperation gradually emerged when *v*_*y*_ was sufficiently higher than *v*_*x*_. In contrast, Model 3 showed initial cooperation by conformity, often following cooperation by punishment. This transitional dynamics led to two notable changes: punishment-based cooperation emerged over broader normative conditions, and it did more rapidly.

## 4. Discussion

We developed a set of gene–culture coevolutionary models that explore the coevolutionary process of norm psychology and cooperation. Our results confirmed the possibility that a large-scale cooperative society can emerge via norm internalisation with altruistic norms as well as the dynamics underlying coevolution, which is consistent with cultural group selection theories. The evolution of norm psychology can lead to substantial variation between groups, resulting in a large-scale cooperative society. Furthermore, our models allowed us to identify the types of social norms that contribute to the evolution of cooperation through their embodiment by socialised agents, and draw novel connections between different types of social norms and cooperation. In this final section, we highlight the key findings about each social norm and discuss the implications of the results, limitations, and directions for future research.

### 4.1. Summary

#### Injunctive norm

Our study provides insight into the conditions for injunctive norms that favour the coevolution of norm psychology and cooperation: sufficiently encouraging punishment compared with cooperation. This aligns with prior theoretical studies (Gavrilets & Richerson, 2017), highlighting the equilibrium function of punishment that makes any behaviour viable (Boyd & Richerson, 1992). Our analysis reveals how punishment maintains cultural equilibria of cooperation. In a cooperative society underpinned by punishment, individuals are split into two types: vigilantes, who have strongly internalised the punishment norm, and selfish agents, who have only payoff-oriented considerations in decision-making (Figure 3b; see the SI, Figures S3, S4, S6 and S7 for dimorphic populations in genotype *α*_*i*_and phenotypes (*x*, *y*)). In groups with vigilantes, the payoff from defection is less than that from cooperation. Consequently, a uniform cooperative group consisting of vigilantes and conditional cooperators emerge to evade punishment.

More interestingly, the comprehensive manipulation of injunctive norms refined the sufficient conditions for the evolution of cooperation. Strongly encouraging cooperation with injunctive norms tends not to favour the norm psychology and eventual cooperation. This suggests that those internalising cooperative norm may face exploitation by free riders, highlighting the potential drawback of injunctive norms in promoting cooperation – a novel finding in our study.

#### Descriptive norm

Our models also examined the adaptive value of the psychology of internalising descriptive norms (i.e. conformity) in a social dilemma. We demonstrated that conformity is unlikely to evolve from scratch in this domain. Then, we presumed that this domain-general learning capacity was brought into the specific domain of cooperation and work. We showed that this could coevolve with cooperation through the selective force of intergroup competition under this presumption.

However, cooperative societies built upon conformity exhibit different stability features compared with those supported by punishment. Offline simulations, where cooperative groups from online simulations engage in 40 rounds of public goods games again, reveal that these societies are not inherently stable (see the SI, Figure S11, for the online simulation results closest to the centroid of each cluster and the offline simulation results). This instability arises because the emergence and sustainability of cooperative groups hinge on the prevalence of cooperation, and descriptive norms can fluctuate, introducing structural challenges in maintaining cooperation.

#### Interplay between Injunctive and Descriptive norm

In the earlier discussions, we highlighted two potent evolutionary drivers of cooperation: punishment and conformity. Moreover, we argue that exapted conformity might have served as a scaffolding for the evolution of punishment, primarily owing to the differing strength of attraction between punishment- and conformity-based cooperative societies and the great speed of cultural relative to genetic evolution. This dynamic process, which leads to the expansion of the basin of attraction for punishment-based cooperation, unfolds as follows. First, conformist transmission initiates a cultural process that establishes and sustains group boundaries. In a mixed population of cooperative and non-cooperative groups, injunctive norm psychology *α*_*i*_, driving agents to internalise altruistic punishment norms, is likely to be favoured. Thus, with exapted conformity, cooperation evolves under broader normative conditions. In other words, the exaptation assumption can provide a different starting point for the fitness landscape, thereby reaching a higher peak.

This finding partially supports the argument that punishment and conformity played complementary roles in the evolution of prosociality (Henrich & Boyd, 1998, 2001; Andresguzman et al., 2007). However, it does not align with the prediction that two micro mechanisms would work and evolve together in social dilemmas, either culturally (Henrich & Boyd, 2001) or genetically (Andresguzman et al., 2007). Whether the model allows for both cultural and genetic evolution may account for the inconsistency between such arguments and the findings of this study. Our results suggest that, given the assumption that agents acquire learning biases genetically and behaviours culturally, the coexistence of conformity and punishment is unlikely or short-lived. Instead, as elucidated above, each fosters distinct forms of cooperative societies, with macro transitions between them.

### 4.2. Implications

Our theoretical predictions align with existing empirical evidence, emphasising the pivotal role of the punishment norm in human cooperation. Human proclivity for third-party punishment in response to norm violations has been well documented (Fehr & Fischbacher, 2004; Henrich et al., 2006; Mathew et al., 2013). This study, which demonstrated the spontaneous emergence of vigilantes who punish willingly at a cost, provides an explanation for those dispositions. As theoretically suggested (Akçay & van Cleve, 2021), the internalisation of external punishments by individuals could form a more stable foundation for social order based on punishment, potentially evolving into formalised institutions like law enforcement (North, 2010).

Conformity, according to our predictions, plays a crucial role in the evolutionary process of cooperation, particularly in environments with high migration rate parameter *m* = 0.5, which exceeds the observed migration rates among actual hunter–gatherer populations (Marlowe, 2005). Theoretically, the increase in migration reduces not only genetic but also cultural differences among groups, thus making the condition for the evolution of cooperation more stringent. However, in our models, conformity mitigates the condition (see the SI, Figures S15 and S16, for a summary graph with smaller and larger migration rates, *m*). Nonetheless, conformity by itself lacks the ability to establish a robust order and is susceptible to negative selection over time. This aligns with the theoretical preference for ‘weak conformity’ in previous studies (Claidière et al., 2012; Kandler & Laland, 2009), which has empirical support (Eriksson & Coultas, 2009; McElreath et al., 2005). Taken together, the two proximate mechanisms maintaining social norms and resulting normative regularities provide clues as to the framework in exploring the potential of non-human norms and interpreting empirical data (Andrews et al., 2024).

### 4.3. Limitations and future directions

However, the conclusions drawn from our simulations warrant caution owing to some impactful assumptions on results. A key assumption involves intergenerational strategy transmission, where we conservatively posit random strategy acquisition at the beginning of each generation. If, alternatively, we assume vertical transmission from parents, the dominance of conformity in maintaining uniformity proves too strong for cooperation to emerge once defection stabilises (see the SI, Figure S12, for a summary graph with a vertical transmission setting). This finding is consistent with previous studies asserting that strong conformity can impede the spread of adaptive variants (Kandler & Laland, 2009).

Moreover, our model relies on several assumptions regarding social norms. First, we represented injunctive and descriptive norms as independent, following Cialdini et al. (1990), although they can be viewed as almost identical or strongly related. However, real-world observations indicate that descriptive and injunctive norms can sometimes be incongruent (Cialdini et al., 1991; Ewing, 2001; Perkins & Berkowitz, 1986). Although this assumption led to novel findings, it concurrently introduces a limitation: the model does not consider the endogeneity of injunctive norms, avoiding potential confounding effects arising from two endogenous norms. Of course, some argue in favour of this assumption, positing that, in pre-modern societies, norms were external rules not generated within the society, implying that injunctive norms were not subject to endogenous evolution (Giddens, 1991). However, historical events, such as the Reformation, highlight conflicts between societies with different injunctive norms significantly affecting behaviours and beliefs. Future studies are essential to explore the selection process among societies with endogenous and evolving injunctive norms shaped through continuous social interactions. Secondly, for simplicity, we assumed that the norm psychologies underlying social norms were invariant within generations. However, empirical evidence suggests systematic interplay between two types of norm psychologies over cultural time (Bicchieri, 2005; Bicchieri & Xiao, 2009). The observed transition from descriptive to injunctive norms over evolutionary timescales in this study may potentially occur in a developmental process (Heyes, 2023). This will contribute to a more nuanced understanding of how different types of social norms coexist and influence human behaviours.

### 4.4. Conclusion

This study explores the prospect of socialisation by humans even under altruistic norms. Sociologists argue that behind large-scale human cooperation lie norms that embody common values and restrain self-interested behaviour, treating agents as social actors shaped by norm internalisation. However, criticisms of the teleological nature of the ‘over-socialised’ concept have prompted a deeper exploration of the functional significance of internalising social norms. Thus, this study bridges sociology, economics and biology to scrutinise the validity of socialisation theory. Addressing the initial question posed in this study, it is now conceivable that humans can indeed be socialised into prosocial norms. This study yields two key insights. First, injunctive norms that prioritise punishment over cooperation prompt internalisation, fostering the evolution of cooperation. Second, the psychological mechanism of internalising descriptive norms may establish the prerequisites for a large, cooperative society sustained by punishment. These findings contribute to a multidisciplinary understanding of human social dynamics, shedding light on the nuanced interplay between individual psychology, social norms and cooperative behaviour.

## Supporting information

Kido and Takezawa supplementary material Kido and Takezawa supplementary material

## Data Availability

n/a.

## Supplementary material

To view supplementary material for this article, please visit https://doi.org/10.1017/ehs.2024.37

## Research transparency and reproducibility

All simulation codes are available at https://github.com/kidd000/Kido_Takezawa2024.
