# Supplementary material for: Coevolution of norm psychology and cooperation through exapted conformity
Source: Evol Hum Sci. 2024 Oct 24;6:e35. doi: 10.1017/ehs.2024.37 (PMC11503932; doi:10.1017/ehs.2024.37)
Supplement: Kido and Takezawa supplementary material [file S2513843X24000379sup001.docx]

Supplementary Materials
Coevolution of norm psychology and cooperation under exapted conformity

# Supplementary Models

[**Text S1.1 Endogenous Parameters** 2](#_Text_S1.1_Endogenous)

[*Initial values of norm psychology in the 1st generation* 2](#_Toc153735498)

[*Initial values of strategy in the 1st round of each generation* 2](#_Toc153735499)

[**Text S1.2 Exogenous Parameters** 3](#_Toc153735500)

[*Manipulated parameters* 3](#_Toc153735501)

[*Constant parameters* 3](#_Constant_Parameters)

[**Text S1.3 Additional information** 4](#_Toc153735503)

[*Strategy and material payoff* 4](#_Toc153735504)

[*Strategy revision algorithm* 5](#_Toc153735505)

[*Multi-level selection algorithm* 6](#_Toc153735506)
[*Formulation of the fixation index (*$F_{ST}$*)* 6](#_Formulation_of_the)

Table S1: Parameters.

## Text S1.1 Endogenous Parameters

We placed the genotypic and phenotypic traits possessed by the agents as endogenous variables. As genotypic traits, we considered the norm psychology of injunctive norms $\alpha_{i}$ and the norm psychology of descriptive norms $\alpha_{d}$. These values remain constant during the individual’s life; they are subject to random mutations when the trait is inherited from the parent to the offspring (i.e., during reproduction). The mutation is modeled as follows:

$$\alpha^{'}=\alpha+\lambda$$

where $\lambda$ is a Gaussian noise added to $\alpha$ with probability $\mu=0.0001$ per offspring, following the normal distribution with mean zero and standard deviation $0.1$. Note that norm psychology after mutation $\alpha'$ can never be $\alpha'<0$ or $\alpha' >1$, in which case gaussian noise will be regenerated until $\alpha'$ falls within the range $[0,1]$.

As phenotypic traits, we considered cooperative behavior $x$ and punishment behavior $y$. These strategies are modified by the agents according to the myopic optimization protocol with their utility functions (see Text S1.3 for more detailed algorithm). Descriptive norms also changed dynamically because they were perceived as the frequency of others’ behaviors at each time step in a group.

### Initial values of norm psychology in the 1st generation

Initial values of both $\alpha_{i}$ and $\alpha_{d}$ were taken randomly from a uniform distribution $U(0, 0.05)$, respectively. However, with the assumption of exapted $\alpha_{d}$, the initial values of $\alpha_{d}$ were taken randomly from the following distributions: $N\left( 0.1, 0.25 \right), N\left( 0.3, 0.25 \right)$and $N(0.5, 0.25)$. Among these, in the main text, we report the results based on the assumption of $N(0.3, 0.25)$ as the initial distribution. For results under other assumptions, see Figure S1.

### Initial values of strategy in the 1st round of each generation

The initial strategies $(x,y)$ of individuals at the beginning of each generation are assigned randomly with equal probabilities. Thus, offspring’ behaviors at the beginning of their lives were acquired independent of their parents' strategies. However, we can also assume a cultural process in which behavioral traits are vertically transmitted from parents, not only genetic traits. For results under this assumption, see Figure S12.

## Text S1.2 Exogenous Parameters

### Manipulated parameters

Among exogenous parameters, we examined their impact on the evolution of endogenous parameters, especially norm psychology and cooperation, by manipulating the values of the following parameters: injunctive norm values for cooperation ($v_{x}$) and punishment ($v_{y}$), group size parameter ($n$), and migration rate parameter ($m$). The results reported in the main text were performed 25 times for each parameter combination, and for the Supplementary Results, 25 times for the simulations under the setting of vertical transmission and 10 times for the others (i.e., simulations under other group size and migration rate parameters). We exogenously gave values varying from 0 to 1 by 0.1 as the strength of normative values $v_{x}$ and $v_{y}$; gave {8, 16, 24} as group size $n$; gave {0.25, 0.5, 0.75} as migration rate $m$. As for the parameters $n$ and $m$, in the main text, we report the results under the setting $n=16$ and $m=0.5$. For results under other assumptions, see Figure S5-6 ($n$); Figure S7-8 ($m$).

### Constant parameters

We placed the following variables as constant parameters: number of generations $T=30,000$; number of groups $G=500$; number of rounds per generation $Q=40$; benefit of cooperation $b=4$; cost of cooperation $c=1$; cost of punishing $p=n/16$; cost of being punished $q=3p$; cost of monitoring $c_{mon}=n/160$; probability of strategy revision $r=0.25$; error rate of strategy revision $e=0.05$. Note that the above implies that the costs of punishing, of being punished, and of monitoring groupmates increase linearly with group size $n$.

In addition, note that the settings in this study were different from those in Gavrilets and Richerson (2017), on which our models were built, in that we did not consider the cost parameter of optimization ($c_{opt}$) and internalization ($c_{int}$). In Gavrilets and Richerson (2017), these parameters were set as the cost of biological fitness, but not any relative cost since the values of the two parameters were equal ($c_{opt}$=$c_{int}$=0.05). Thus, they only result in a decrease in the baseline fitness parameter from 1 to 0.95, so we removed them. Correspondingly, the result is slightly different from that of Gavrilets and Richerson (2017), but no qualitative difference is observed.

## Text S1.3 Additional Information

### Strategy and material payoff

Consider a situation in which individuals can choose whether to cooperate ($x=1$) and whether to punish ($y=1$). This is a class of collective action problems that Gavrilets and Richerson (2017) referred to as "us versus nature" games. All group members equally receive the following benefits of cooperation pooled in a group according to the number of cooperators:

$$\begin{aligned} \pi_{CA}=b\cdot P\#\left( S1 \right) \end{aligned}$$

Here, $b$ is the benefit parameter of collective action and $P$ is represented by $X/(X+n/2)$, where $X =\sum_{1}^{n} x$, which is the frequency of cooperators in a focal group. That is, $P$ gives the normalized value of cooperation benefits that increases with the number of cooperators in a group of constant size $n$.

Then, depending on each agent’s strategy $(x,y)$, the cost was subtracted from $\pi_{CA}$, resulting in the following material payoff for an individual.

$$\begin{aligned} \pi\left( x,y \right)=\pi_{CA}-x\cdot c-y\cdot\left[ \left( 1-\tilde{X} \right)\cdot p+c_{mon} \right]-\left( 1-x \right)\cdot q\cdot\tilde{Y}\#\left( S2 \right) \end{aligned}$$

Here, the positive constants $c$, $p$, $c_{mon}$ and $q$ capture the costs of cooperation, punishment, monitoring defectors for punishment and being punished, respectively (Table S1). $\tilde{X}$ and $\tilde{Y}$ are the frequencies of each strategy among group members other than a focal individual. That is, in Eq. S2, the strategic cost, consisting of the cost of cooperation (the second term), the cost of punishment (the third term), and the cost of penalty suffering from punishment (the fourth term), is subtracted from the benefits of collective action $\pi_{CA}$ (the first term), which amounts to an individual payoff (Figure S1-1). Note that cooperation costs a constant value, whereas the cost of punishment increases with the frequency of defectors in a group (i.e., $1-\tilde{X}$) and the cost of being punished increases with the frequency of punishers in a group (i.e., $\tilde{Y}$). For a sensitivity analysis of the parameters related to the payoff structure (e.g., the benefit parameter of collective action $b$ and the cost parameter of cooperation $c$), refer to the supplementary material of Gavrilets and Richerson (2017).

Figure S1-1: Payoff function for the focal individual’s strategy $(x,y$) in relation to the numer of cooperators in a group other than the focal individual when group size $n=8$ (left column), $n=16$ (middle column), and $n=24$ (right column). Shown in the first row are focal individuals’ payoffs when 0% of group members are punishers, and in the second row are ones when 50% of members are punishers. Other parameters are the same in the main text: $b=4, c=1, p=n/16,q=3p, c_{mon}=n/160$.


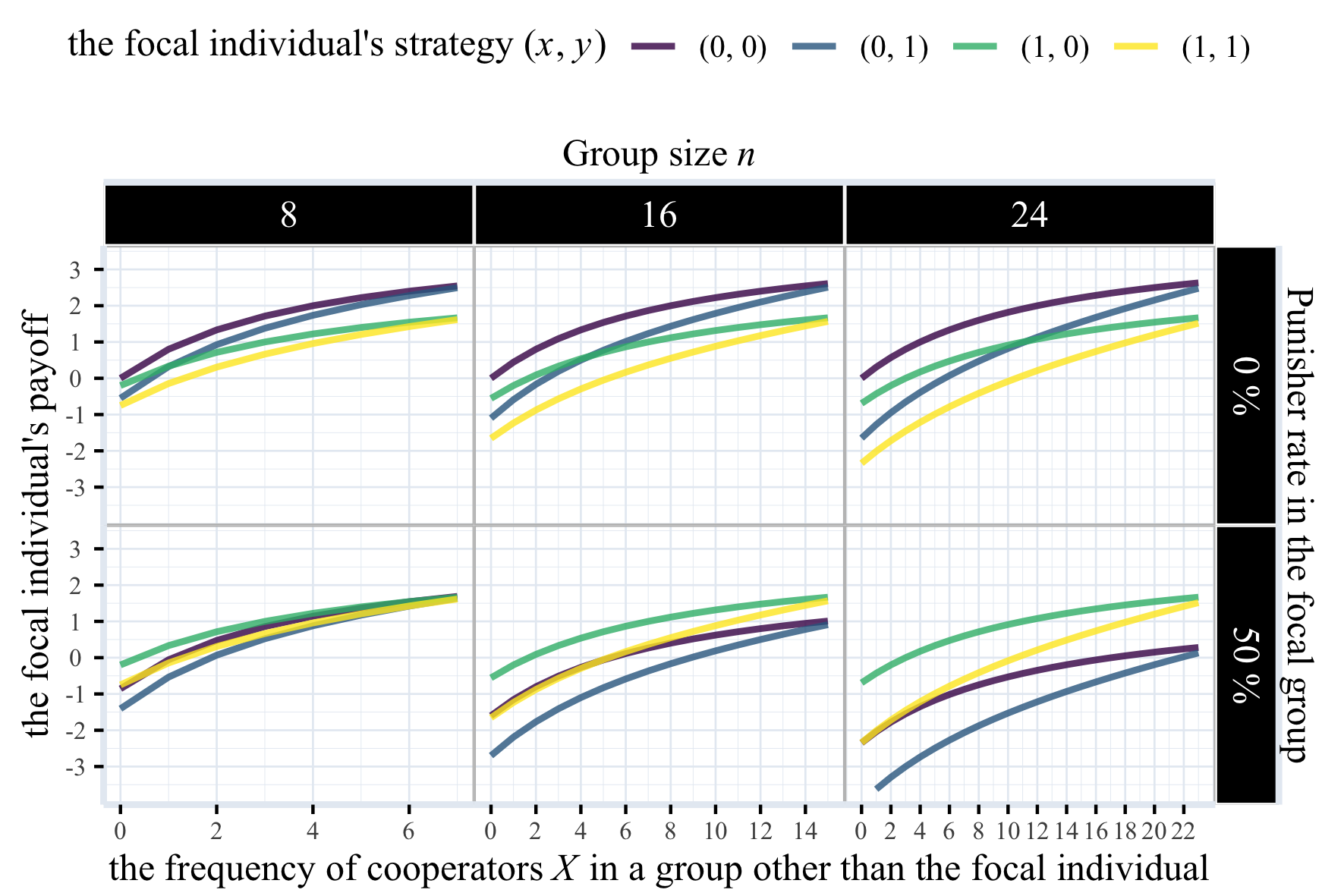


### Strategy revision algorithm

To simulate the best response dynamics in an environment where group members repeatedly engage in a collective action with punishment, we adopted the following algorithm of myopic optimization, as in Gavrilets and Richerson (2017). First, given the behaviors of the other group members in the previous round, individuals calculate how much utility they would have obtained had they adopted each of the four total strategies. Here, utility depends on three influences: payoff, injunctive norm, and descriptive norm (Eq. 1 in the main text). Then, the genetic parameters of norm psychologies give the parameters weighting each factor and individuals update to $arg\max_{x,y} u_{\alpha_{i}\alpha_{d}}(x, y)$ with probability $r=0.25$ each round.

$\begin{aligned} \begin{aligned} \mu_{\alpha_{i}\alpha_{d}}\left( x,y \right)&=\frac{\left( 1-\alpha_{i} \right)\left( 1-\alpha_{d} \right)}{\left( 1-\alpha_{i} \right)\left( 1-\alpha_{d} \right)+\alpha_{i}+\alpha_{d}}\cdot\pi\left( x,y \right) \\ &+\frac{\alpha_{i}}{\left( 1-\alpha_{i} \right)\left( 1-\alpha_{d} \right)+\alpha_{i}+\alpha_{d}}\cdot\left( v_{x}\cdot x+v_{y}\cdot y \right) \\ &+\frac{\alpha_{d}}{\left( 1-\alpha_{i} \right)\left( 1-\alpha_{d} \right)+\alpha_{i}+\alpha_{d}}\cdot\left[ \tilde{X}\cdot x+\left( 1-\tilde{X} \right)\left( 1-x \right)+\tilde{Y}\cdot y+\left( 1-\tilde{Y} \right)\left( 1-y \right) \right]. \end{aligned}\#\left( S3 \right) \end{aligned}$Agents with high $\alpha_{i}$ (norm psychology of injunctive norm) have a greater weighting in the second term and obtain higher utility from following the injunctive norm. Agents with high $\alpha_{d}$ (norm psychology of descriptive norm) give more weight to the third term and obtain higher utility from conforming to others. Note that an optimization error occurs with probability $e=0.05$, and the agent updates to a random combination of strategies. Individuals not updating their strategies keep their strategies in the next round.

### Multi-level selection algorithm

Selection on norm psychology $\alpha_{i}, \alpha_{d}$ follows the two-level Wright-Fisher process, as in Gavrilets and Richerson (2017). The number of groups $G$, group size $n$, and thus population size remained constant across all generations. The overall process involved individual-level selection followed by group-level selection. First, in group-level selection, group fitness $w_{j}$ is given by the cumulative number of cooperators across $Q$ rounds in a group.

$$\begin{aligned} w_{j}=\sum_{1}^{Q} \sum_{1}^{n} x\#\left( S4 \right) \end{aligned}$$

Group fitness $w_{j}$ determines the survival and replication rates of each group. Second, in individual-level selection, individual fitness $w_{i}$ is given by the addition of baseline fitness $w_{0}\left( =1 \right)$ and a mean of the payoffs over $Q=40$ rounds, $\bar{\pi}_{i}$.

$$\begin{aligned} w_{i}=w_{0}+\bar{\pi}_{i}\#\left( S5 \right) \end{aligned}$$

Individual fitness $w_{i}$ determines the survival and reproduction rate of each individual. Offspring born through individual-level selection are subject to mutation that causes noise in their genetic traits, the norm psychology parameters, inherited from their parents (details are discussed in Text S1.1).

### Formulation of the fixation index ($F_{ST}$)

$F_{ST}$ is originally a measure of genetic differentiation between populations (Cavalli-Sforza *et al*., 1994). Formally, $F_{ST}$ is the ratio of between-group variance and total variance. In our study, we calculate $F_{ST}$ as follows:

$$\begin{aligned} F_{ST}=\frac{var_{j\in\mathcal{G}}\left( p_{j} \right)}{\bar{var}_{i\in\mathcal{n}}\left( p_{ij} \right)+var_{j\in\mathcal{G}}\left( p_{j} \right)}\#\left( S6a \right) \end{aligned}$$

where

$$\begin{aligned} var_{j\in\mathcal{G}}\left( p_{j} \right)=var_{j\in\mathcal{G}}\left( \frac{\sum_{i=1}^{n} p_{ij}}{n} \right)\#\left( S6b \right) \end{aligned}$$

$$\begin{aligned} \bar{var}_{i\in\mathcal{n}}\left( p_{ij} \right)=\frac{\sum_{j=1}^{G} var_{i\in\mathcal{n}}\left( p_{ij} \right)}{G}\#\left( S6c \right) \end{aligned}$$

Here, $var_{i\in\mathcal{n}}\left( p_{ij} \right)$ represents variance between trait values $p_{ij}$ for individual $i$ in the set $\mathcal{n=\{}i_{1},\ldots,i_{n}\}$ within a group $j$, and $var_{j\in\mathcal{G}}\left( p_{j} \right)$ represents variance between mean trait values $\frac{\sum_{i=1}^{n} p_{ij}}{n}$ for group $j$ in the set $\mathcal{G=\{}j_{1},\ldots,j_{G}\}$ within a population ($G$ and $n$ are parameters for the number of groups and group size, respectively). We can consider $F_{ST}$ as a measure of the degree of non-randomness regarding the interaction partners of a group. Higher values of $F_{ST}$ increase the probability that interaction partners have similar genetic and phenotypic traits when they are randomly selected from the same group rather than the entire population.

All simulation codes are implemented in Python and available at https://github.com/kidd000/Kido_Takezawa2024.

### **References**

Cavalli-Sforza, L. L., Menozzi, P., & Piazza, A. (1994). The history and geography of human genes. Princeton University Press.

Gavrilets, S., & Richerson, P. J. (2017). Collective action and the evolution of social norm internalization. *Proceedings of the National Academy of Sciences*, *114*(23), 6068–6073.

# Supplementary Result

[*Figure S1: Additional graphs for main result (*$n=16, m=0.5$*)* 8](#_Toc153735510)

[*Figure S2-4: Additional graphs for example of evolutionary dynamics under the setting of non-exapted* $\alpha$ *(Model 2) with* $\left( v_{x},v_{y} \right)=\left( 0.5,0.5 \right)$ 9](#_Toc153735511)

[*Figure S5-7: Additional graphs for example of evolutionary dynamics under the setting of exapted* $\alpha d$ *(Model 3) with* ${(v}_{x,}v_{y})=(1.0, 0.5)$ 11](#_Toc153735512)

[*Figure S8-11: Additional graphs for clustering* 13](#_Toc153735513)

[*Figure S12: Result in the setting of vertical transmission between generations* 17](#_Toc153735514)

[*Figure S13-14: Effect of group size* $n$ 19](#_Toc153735515)

[*Figure S15-16: Effect of migration rate* $m$ 22](#_Toc153735516)

### Figure S1: Additional graphs for main result ($n=16, m=0.5$)

Figure S1 provide more complete results of simulations in the main text (Figure 1). From Figure S1, which is a summary plot with the additional two assumptions of exapted $\alpha_{d}$: initial distributions of $N(0.1, 0.25), N(0.5, 0.25)$, it can be seen that reddish areas in the first row of the heat map expand as the column moves to the right. This means that the stronger the assumption of exaptation (i.e., the higher the mean value of the initial distribution of $\alpha_{d}$), the larger the parameter space in which cooperation evolves in the last generation.

Figure S1: Summary result under the same settings as main text. Heatmap of $x$, $y$, $\alpha_{i}$ and $\alpha_{d}$ for different normative values $(v_{x}, v_{y})$, and 5 models. Other parameters: $n=16$, $m=0.5$. Shown are averages based on 25 runs for each parameter combination.


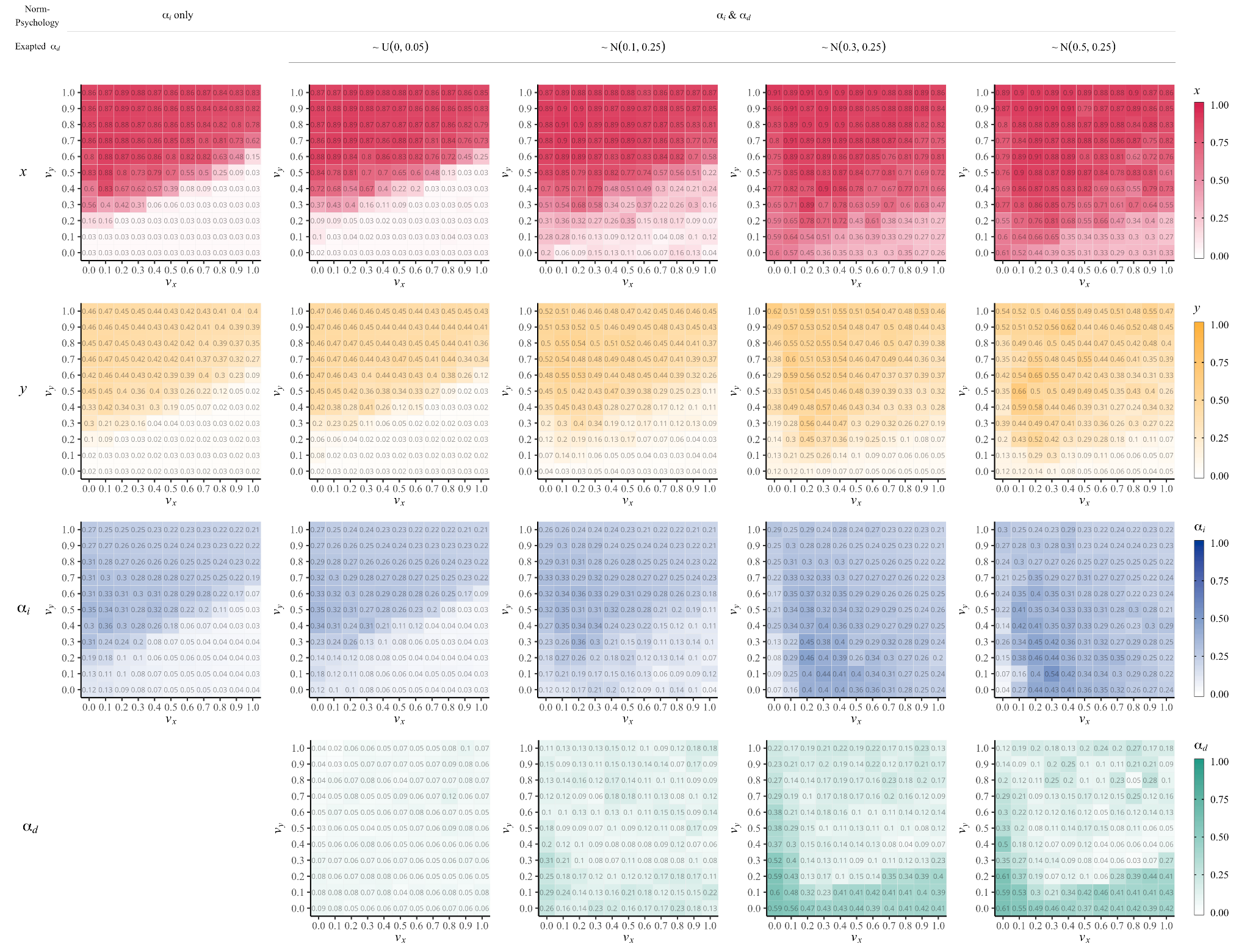


### Figure S2-4: Additional graphs for example of evolutionary dynamics under the setting of non-exapted $\alpha_{d}$ (Model 2) with ${(v}_{x,}v_{y})=(0.5, 0.5)$

From Figure S2 illustrating evolutionary dynamics (main text, Figure 2) for all generations, it can be seen that a steady state of cooperation is maintained after the evolutionary dynamics shown in Figure 2 of the main text. Figure S3 and S4 show the dimorphism in the agents' genetic traits of injunctive norm psychology ($\alpha_{i}$) and the resulting phenotypes consisting of $\left( x,y \right)=(1,1)$, $(1,0)$, and $(0,0).$ Figure S3 captures the individuals’ psychologies across the ternary parameter space at three different time points, 1150^th^, 1250^th^, and 1350^th^ generation, Figure S4.A shows the distribution of genetic traits values in the first and last generations. As discussed in the main text, there appear to be two types of individuals: vigilantes who have high $\alpha_{i}$, and selfish individuals who have low $\alpha_{i}$. These characteristics of the genetic trait yield a steady state with multiple phenotypes, as shown in S4.B, below. Thus, a cooperative group can emerge, consisting of vigilantes whose strategies are $(1,1)$ and conditional cooperators who would take $(1,0)$ with only a few or no vigilantes.

Figure S2: Evolutionary dynamics under the setting of non-exapted $\alpha_{d}$ (Model 2) with ${(v}_{x,}v_{y})=(0.5, 0.5)$ for all generations (as for evolutionary dynamics over specific generations, Figure 2 in the main text). Mean of $\alpha_{i}$ (blue solid), genetic $F_{ST}$ of $\alpha_{i}$ (blue dotted), mean of $\alpha_{d}$ (green solid), cooperation (red solid) and behavioral $F_{ST}$ of cooperation (red dotted) for a representative simulation.


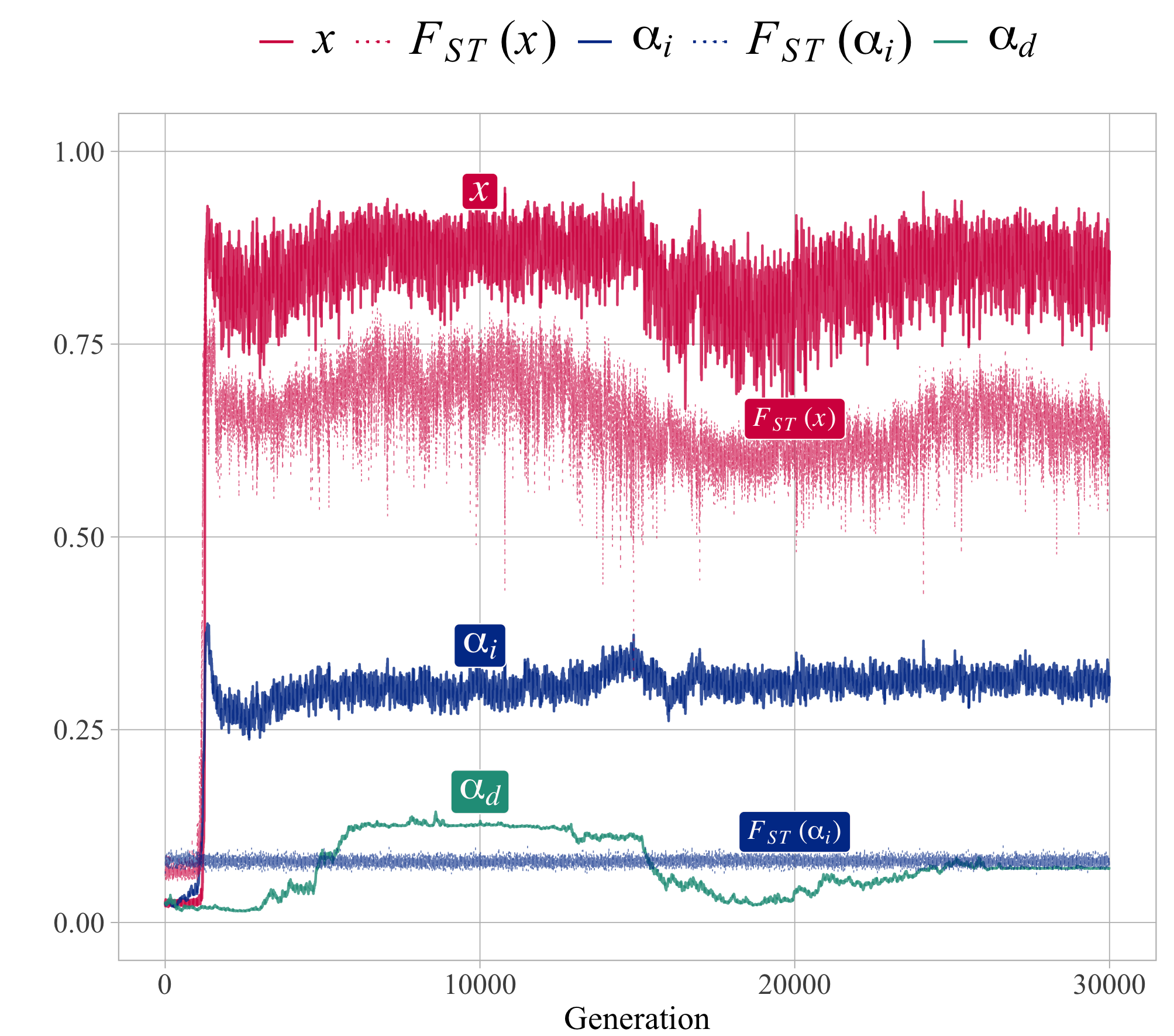


Figure S3: Individuals’ psychologies across the ternary parameter space at three different time points, 1150^th^, 1250^th^, and 1350^th^ generation (as for the evolutionary dynamics and scatter plot of groups on behaviors’ axes during these periods, see Figure 2 in the main text). Each vertex of the triangle corresponds to each preference in an individual’s utility function. The top vertex represents preference for gaining material payoff, corresponding to Eq. $S3a$ in S1.3.The lower left vertex represents preference for following injunctive norm, corresponding to Eq. $S3b$. The lower right vertex represents preference for following descriptive norm, corresponding to Eq. $S3c$. The black point on the diagram represents each individual’s psychology, and the white circle represents the mean psychology in the ternary parameter space.


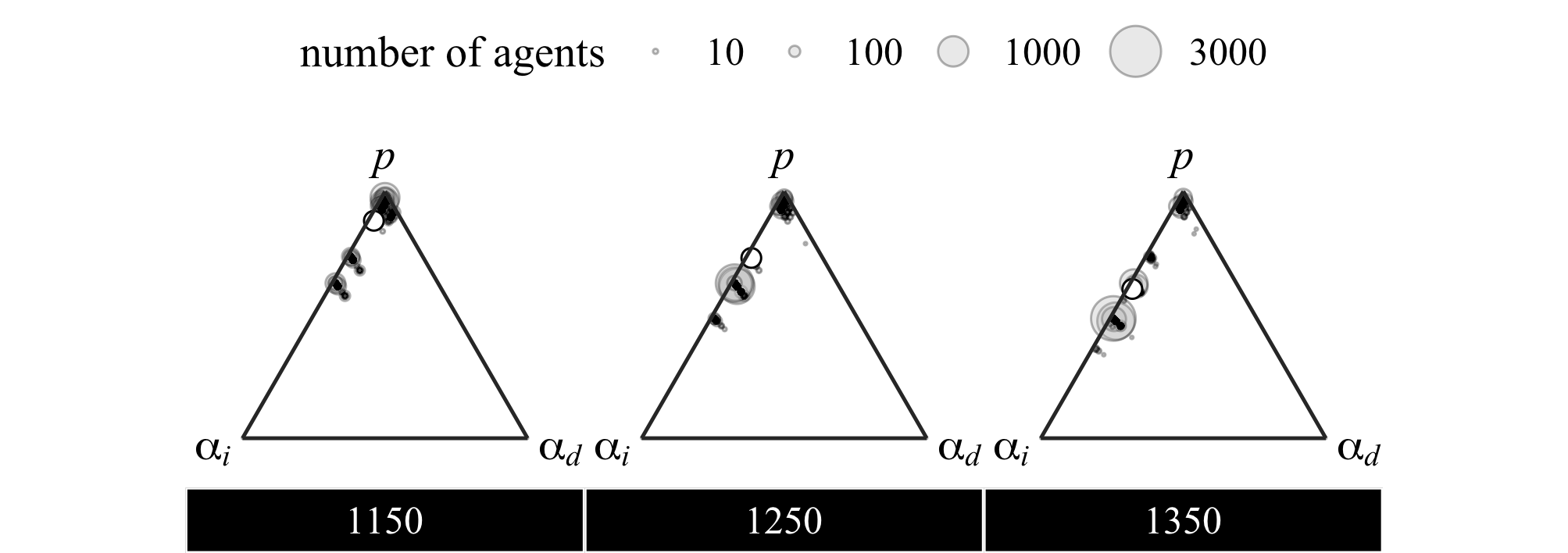


Figure S4: Illustration of the dimorphic population in genotype $\alpha_{i}$ and phenotypes $(x,y)$. (A) Histograms of $\alpha_{i}$ and $\alpha_{d}$ values in the first and last (30,000^th^) generations. (B) Frequency of the combination of strategies $(x,y)$ in the first and last (30,000^th^) generations.


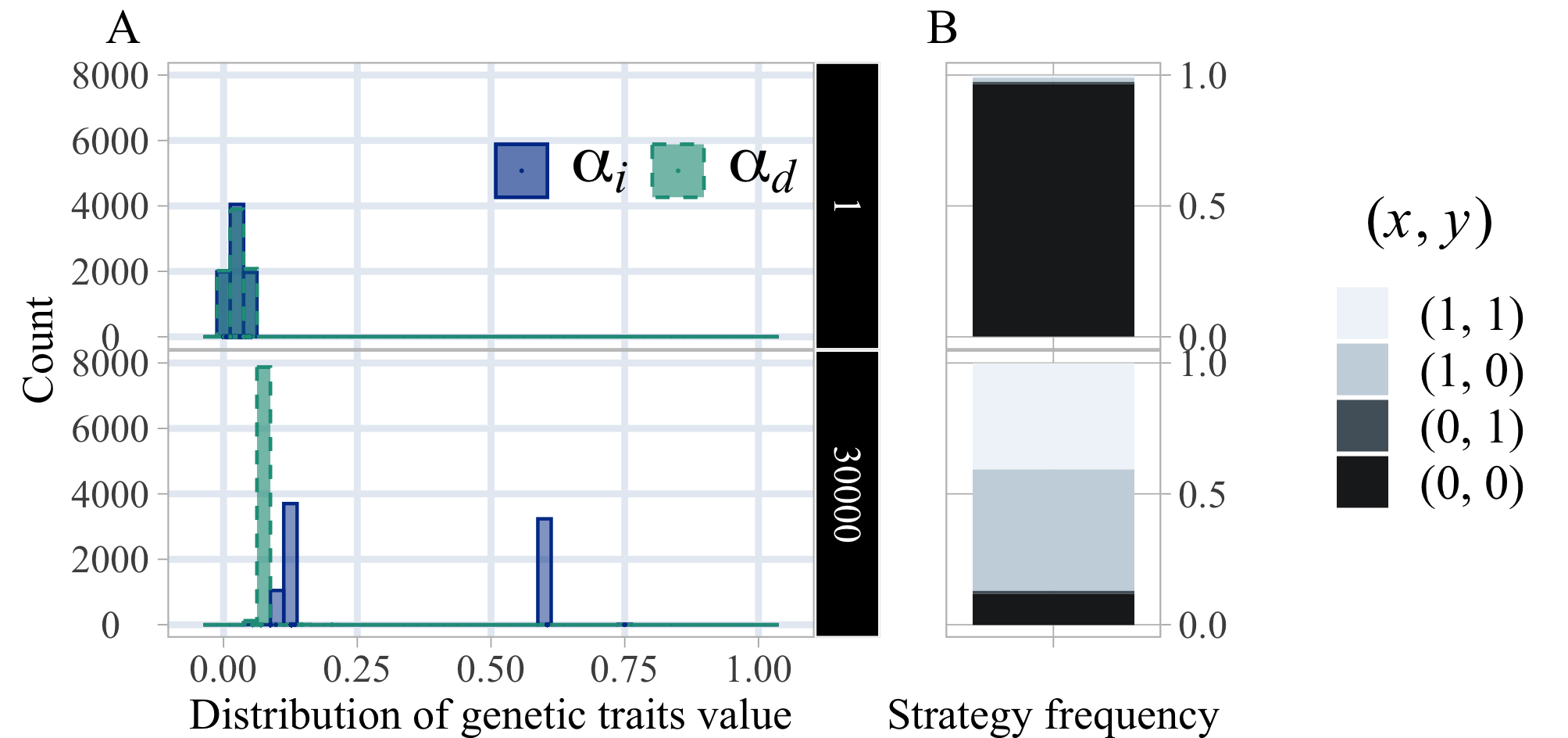


### Figure S5-7: Additional graphs for example of evolutionary dynamics under the setting of exapted $\alpha_{d}$ (Model 3) with ${(v}_{x,}v_{y})=(1.0, 0.5)$

Figure S5 shows the evolutionary dynamics (main text, Figure 4) for all generations. In Model 3 with the assumption of exapted conformity, $\alpha_{d}$ (green line in Figure S5) first evolves from an initial value above 0 to a larger value, and conformity drives cooperation. Next, as $\alpha_{i}$ (blue line) becomes favored and the driver of cooperation takes over, $\alpha_{d}$ steadily declines. These dynamics of genetic traits can be confirmed in Figure S6, which plots individuals’ psychologies across the ternary parameter space in the 300-3200^th^ generations. After the evolutionary dynamics shown in Figure 4 of the main text, a solid cooperative society in which most agents adopt the prosocial strategy ($x=y=1$) lasts for approximately 10,000 generations. Then, the norm psychology undergoes weak negative selection, and finally, as shown in Figure S7.B below, the population is occupied by punishing cooperators ($x=y=1$), mere cooperators ($x=1, y=0$) and defectors ($x=y=0$). The similarity of the steady states of cooperation achieved in Models 2 and 3 can be seen from Figures S4 and S7.

Figure S5: Evolutionary dynamics under the setting of exapted $\alpha_{d}$ (Model 3) with ${(v}_{x,}v_{y})=(1.0, 0.5)$ for all generations (as for evolutionary dynamics over specific generations, Figure 4 in the main text). Mean of $\alpha_{i}$ (blue), $\alpha_{d}$ (green), cooperation (red), and punishment (yellow) for a representative simulation.


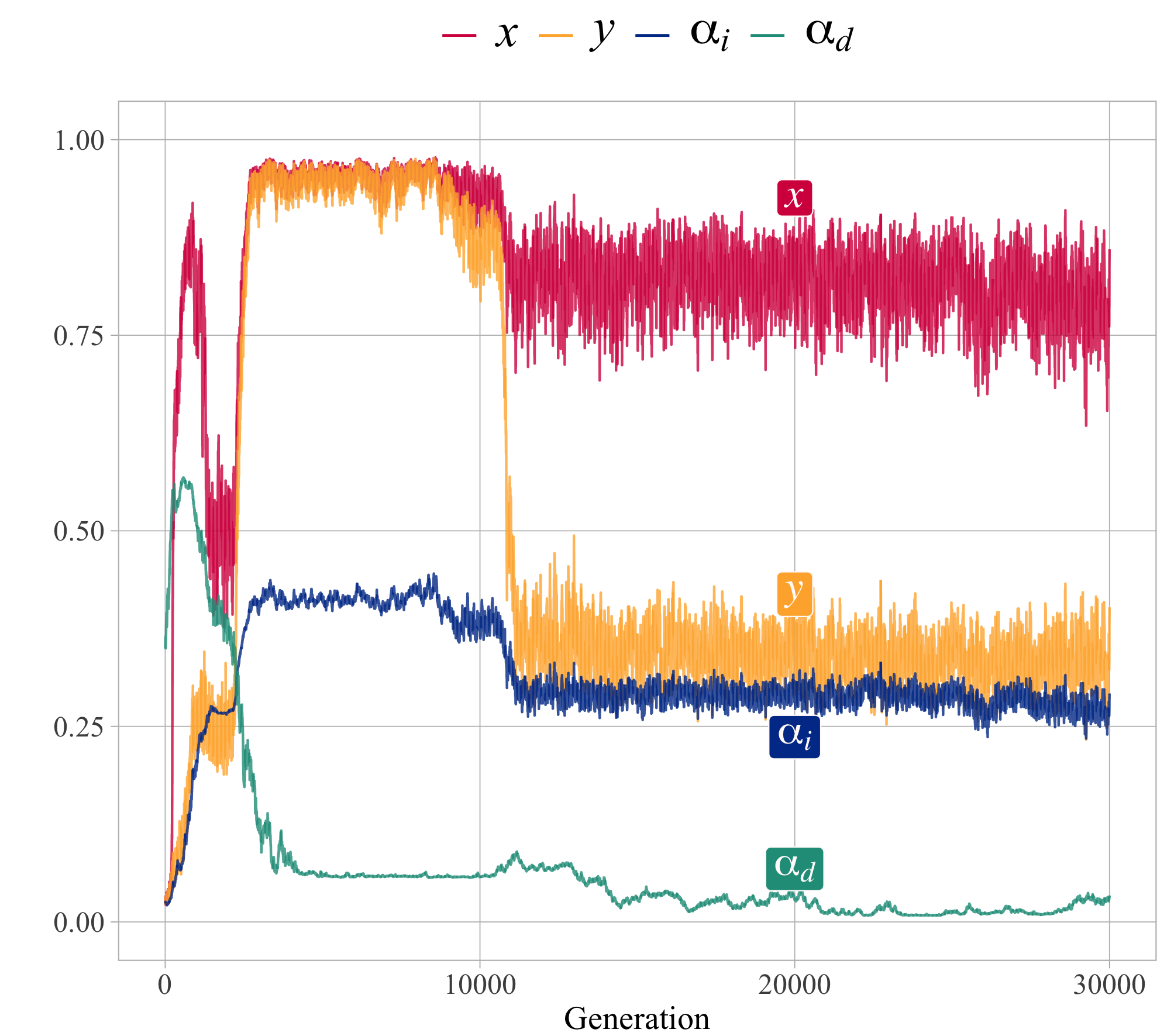


Figure S6: Individuals’ psychologies across the ternary parameter space at three different time points, 1150^th^, 1250^th^, and 1350^th^ generation (as for the evolutionary dynamics and scatter plot of groups on behaviors’ axes during these periods, see Figure 2 in the main text). Each vertex of the triangle corresponds to each preference in an individual’s utility function. The top vertex represents preference for gaining material payoff, corresponding to Eq. $S3a$ in S1.3. The lower left vertex represents preference for following injunctive norm, corresponding to Eq. $S3b$. The lower right vertex represents preference for following descriptive norm, corresponding to Eq. $S3c$. The black point on the diagram represents each individual’s psychology, and the white circle represents the mean psychology in the ternary parameter space.


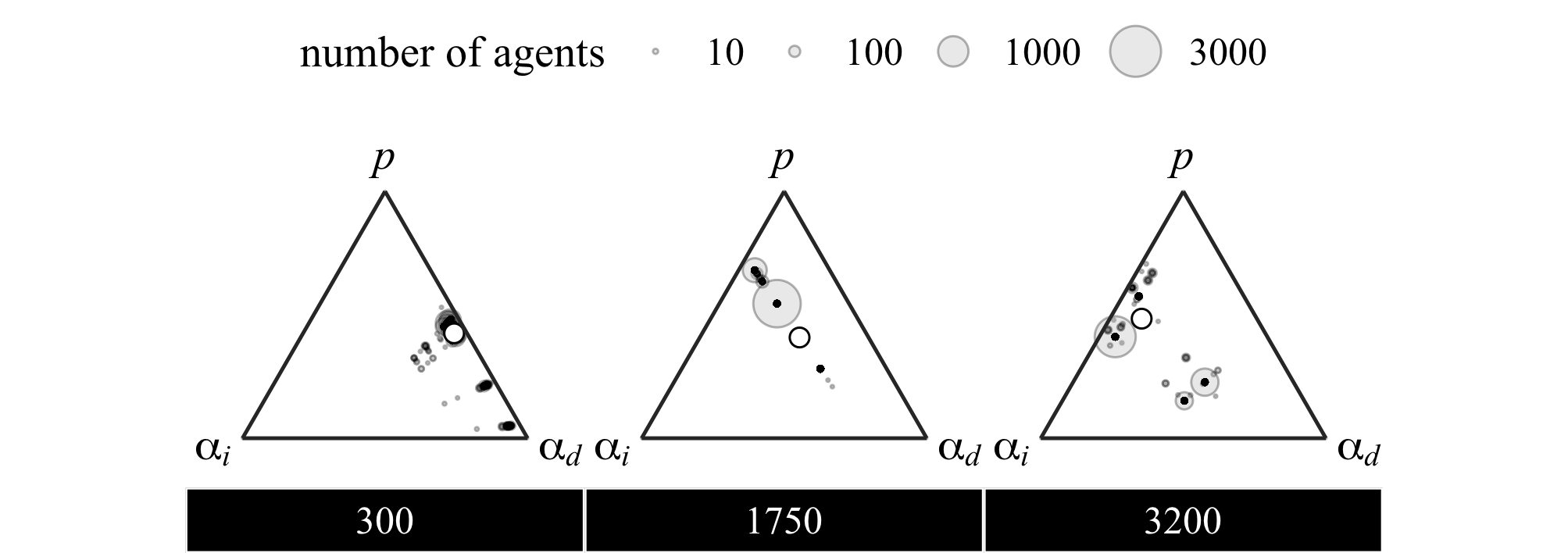


Figure S7: Illustration of the dimorphic population in genotype $\alpha_{i}$ and phenotypes $(x,y)$. (A) Histograms of $\alpha_{i}$ and $\alpha_{d}$ values in the first and last (30,000^th^) generations. (B) Frequency of the combination of strategies $(x,y)$ in the first and last (30,000^th^) generations.


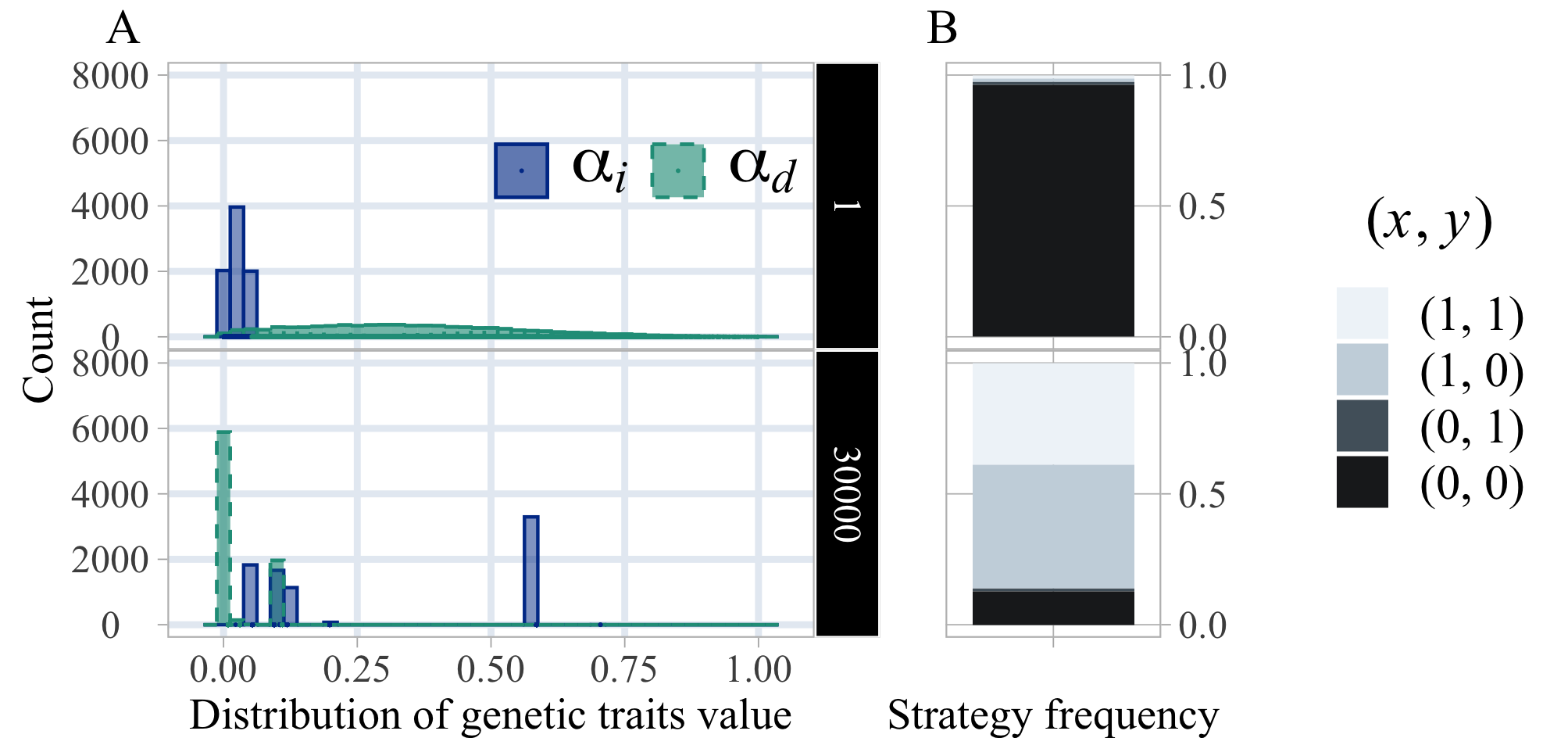


### Figure S8-11: Additional graphs for clustering

Figure S8 shows the clustering analysis for the results of Models 2 and 3. Among them, Figures S8.A-1 and B-1 give the validity for setting the number of clusters to two, and Figures S8.A-2 and B-2 show the clustering distribution mapped on $\alpha_{d}$ and $y$. As also shown in Figure S9, in Model 3 with the assumption of exapted conformity, clusters characterized by high values of $\alpha_{d}$ (i.e., conformity-based societies) appear, and the number of simulations belonging to the cluster characterized by higher punishment rate $y$ (i.e., punishment-based societies) increased in comparison to Model 2. Figure S10, which plots the heatmap of cluster1 rates in Models 2 and 3, also suggests that the basin of attraction of cluster 1 (punishment-based societies) expands dramatically in the parameter space of injunctive norm values ($v_{x}$, $v_{y})$. Figure S11 shows the difference in stability between cooperation achieved by conformity and punishment. Groups that achieved a high cooperation rate through conformity (i.e., cooperative groups in conformity-based societies) in online simulations do not necessarily become cooperative groups when offline simulations are conducted with the same group composition. On the other hand, groups that had achieved a high cooperation rate through punishment (i.e., cooperative groups in punishment -based societies) could maintain a high cooperation rate in offline simulations as well.

Figure S8: Clustering analysis for two models, Model 2 (Left) and Model 3 (Right). (A-1; B-1) Optimal number of clusters $k=2$ based on average silhouette width. (A-2; B-2) Scatterplot of all simulation results for the last generation, with the mean value of $\alpha_{d}$ and the frequency of punishment $y$ on the horizontal and vertical axes, clustered by k-means method with 2 clusters. Results are plotted as yellow circles for cluster 1 and green squares for cluster 2. Each ellipse covers approximately 80% of simulations classified as each cluster, assuming a multivariate normal distribution.


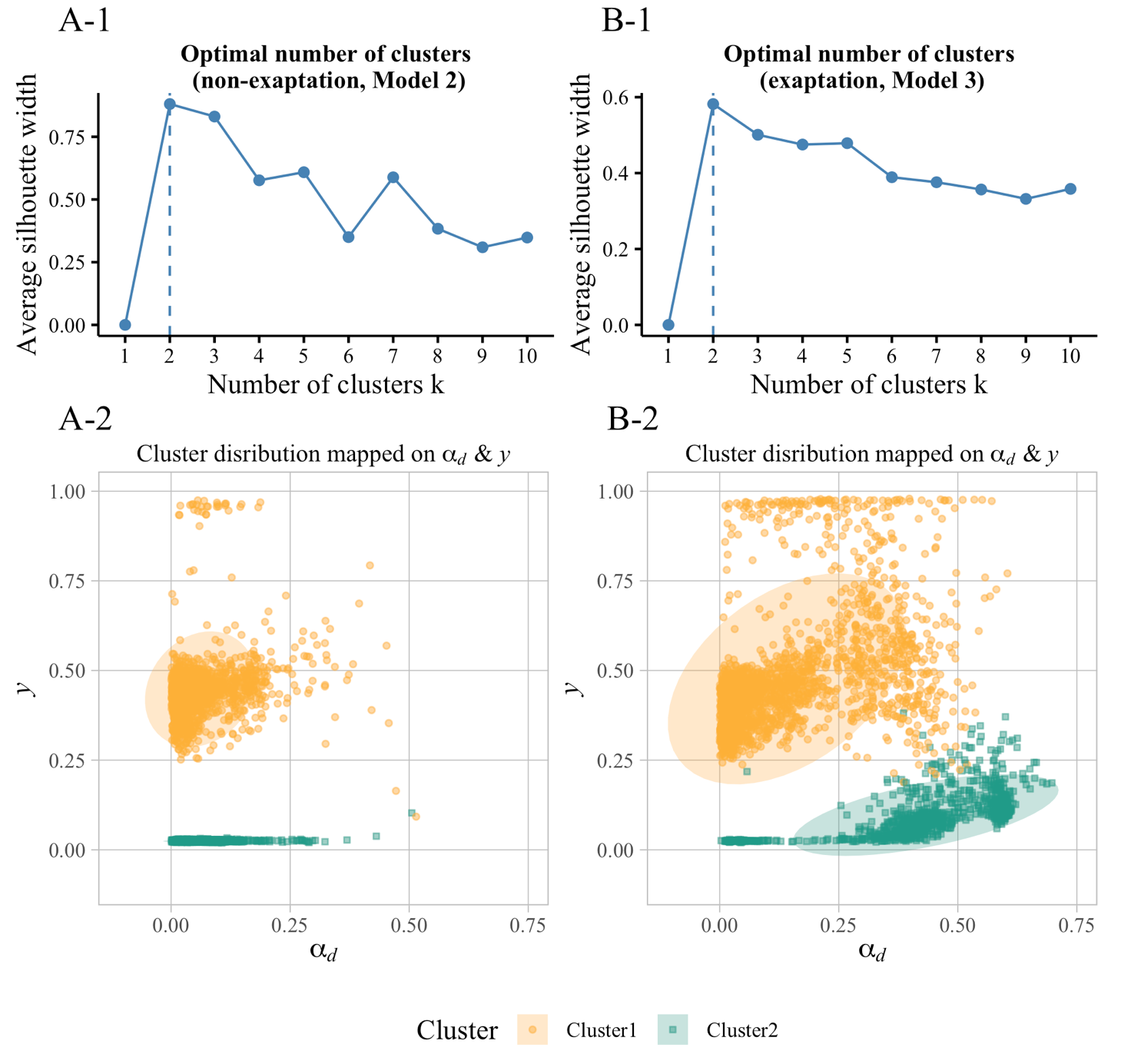


Figure S9: Scatterplot of all simulation results for the last generation on the two dimensions of $\alpha_{d}$ and y for two models, Model 2 (A, Left) and Model 3 (B, Right), with the color representing the cooperation $(x)$ rate for the last generation.


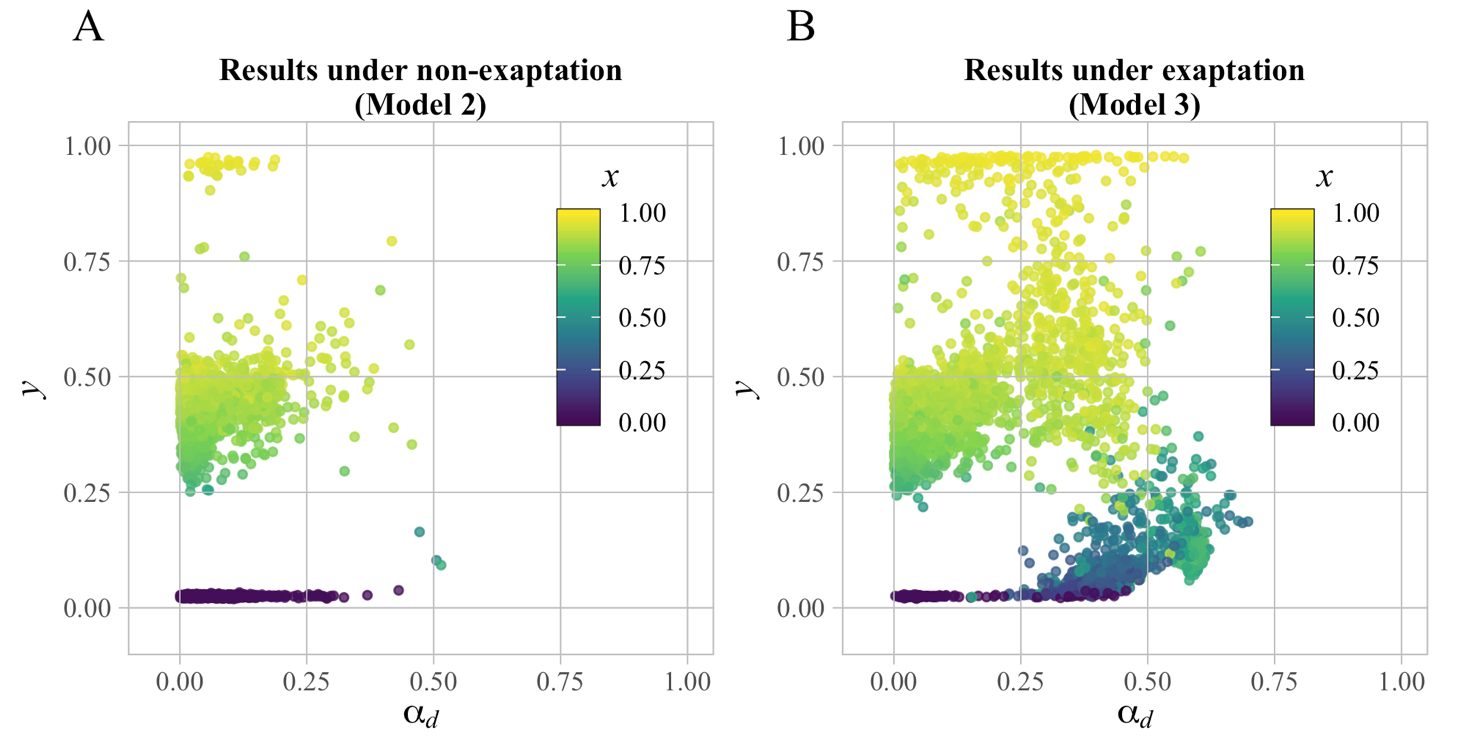


Figure S10: Heatmap of cluster1 rate for each parameter space of injunctive norm values ($v_{x}$, $v_{y})$, for two models, Model 2 (A, Left) and Model 3 (B, Right).


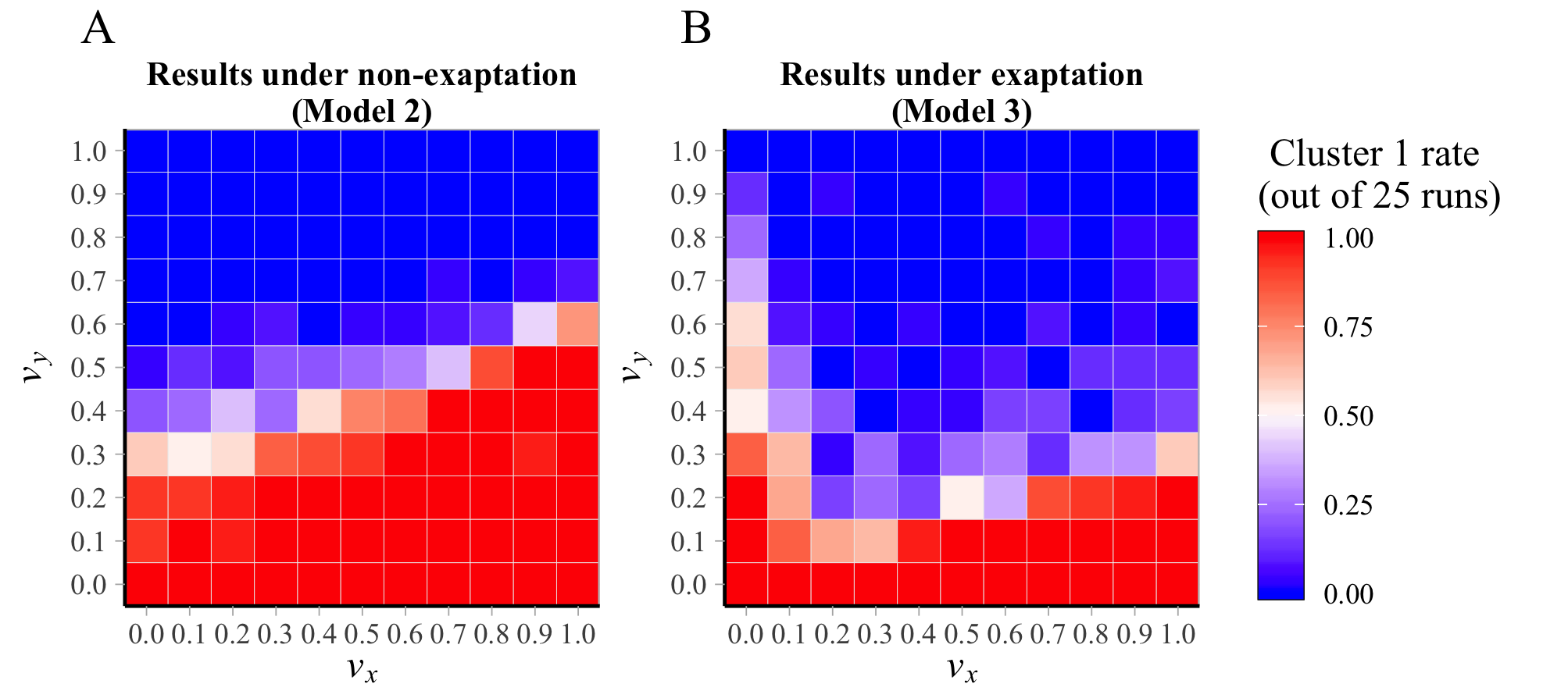


Figure S11. Stability of cooperative groups representing each cluster. (A-1; B-1) Frequency of cooperators and punishers per group for the final generation in the representative online simulation, which is the centroid of each cluster, with the size showing the number of groups whose frequencies of behaviors were the same. The red shaded region represents a group's cooperation rate of 80% or more, and the red dotted line represents the average cooperation rate of groups in the red shade (i.e., groups with a cooperation rate of 80% or more). (A-2; B-2, Left Panel) Line graph showing mean cooperation rates ($x$) over 40 rounds of public goods games with a sample of groups in the red shadow (i.e., offline simulations). The blue shaded region represents mean $x\pm SD$. (A-2; B-2, Right Panel) Histogram of the mean cooperation rates ($x$) of offline simulations for the last round (i.e., 40^th^ round) of each group compared with the online simulations.


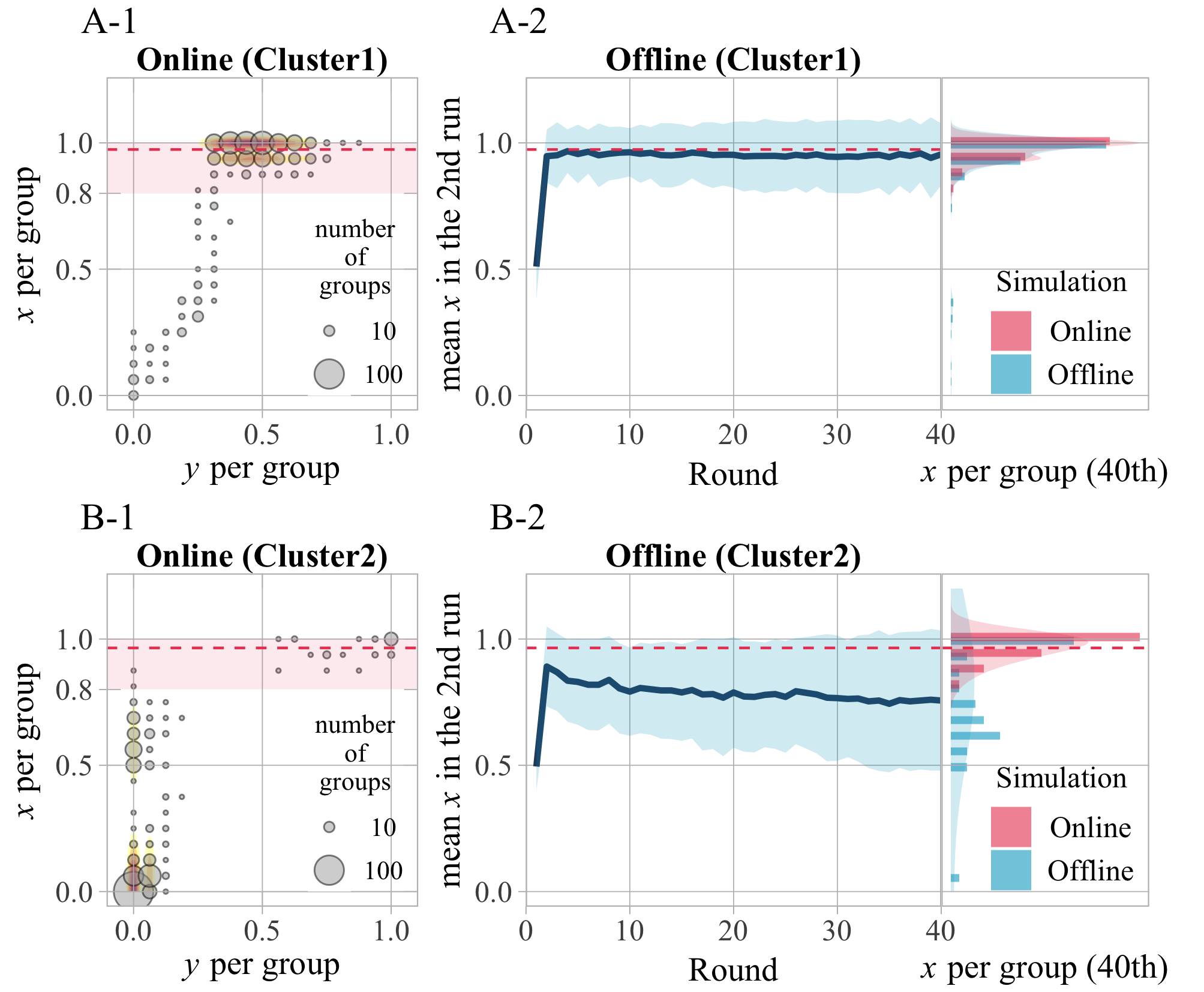


### Figure S12: Results in the setting of vertical transmission between generations

Figure S12 shows the summary plot under the assumption of vertical transmission of phenotypes between generations, which means that the newborn offspring will start life with his parent’s behavioral strategies $\left( x,y \right)$. The simulation results in this assumption are almost qualitatively identical to those reported in the main text (Figure 1 or Figure S1 with more assumptions), but differ only in the following two respects. First, reddish areas in the first row of the heat map do not become large as the column moves to the right, which means that exapted conformity no longer enables cooperation to emerge. As discussed in the main text, this might be due to the backfire of strong conformity that does not allow for a shift from one equilibrium to another. Second, when the injunctive norm encourages cooperation hardly at all (left side of parameter space $(v_{x},v_{y})$for each heatmap), the coevolution of $\alpha_{i}$ and cooperation will not occur.

Figure S12: Summary result under the setting of **vertical transmission between generations**. Heatmap of $x$, $y$, $\alpha_{i}$ and $\alpha_{d}$ for different normative values $(v_{x}, v_{y})$, and 5 models. Other parameters: $n=16$, $m=0.5$. Shown are averages based on 25 runs for each parameter combination.


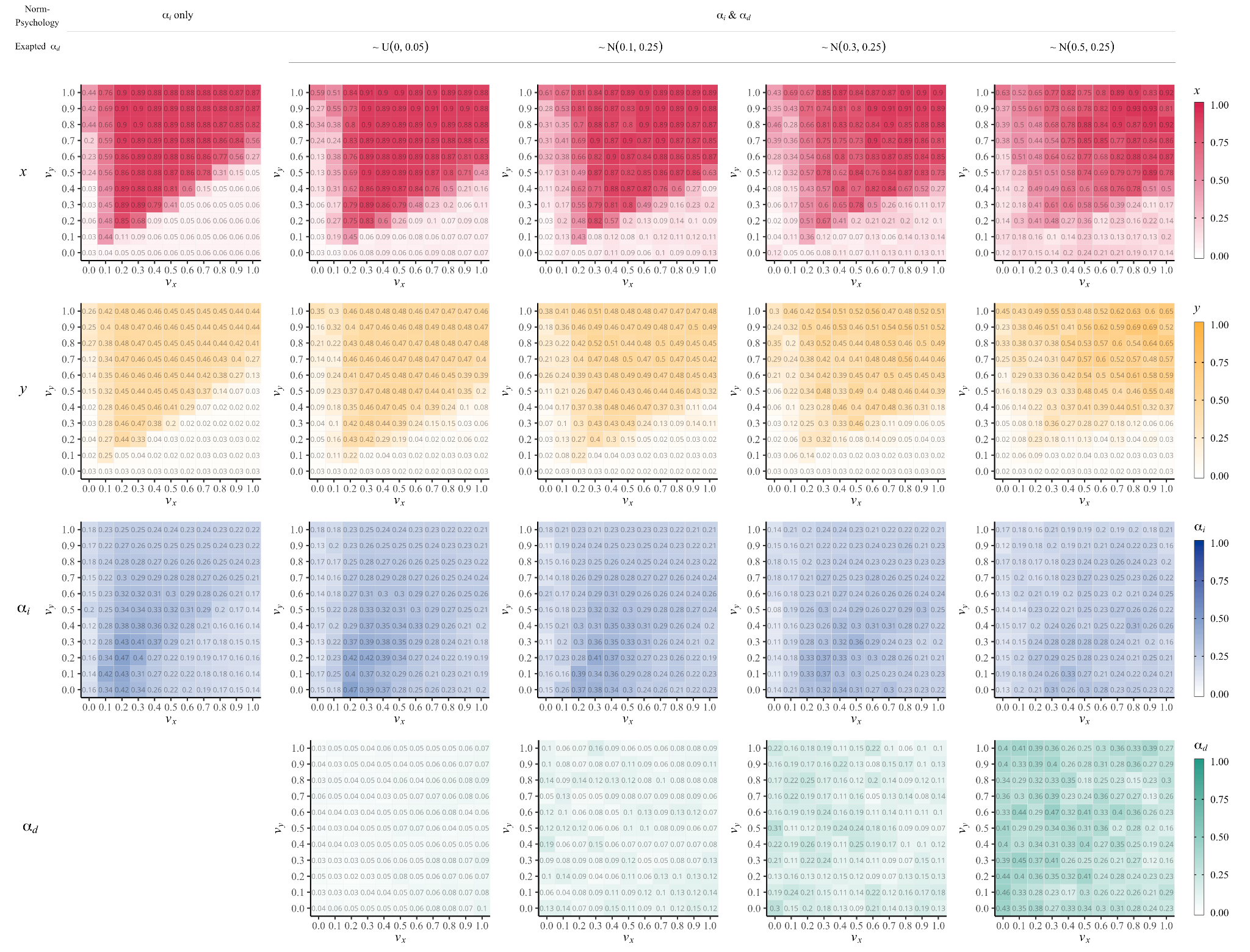


### Figure S13-14: Effect of group size $n$

In the main text, we present the results of an agent-based simulation with the group size parameter $n=16$. Ethnohistorical data from hunter-gatherers living in modern warm climates indicate that the median number of group size is 25.58, which implies that the number of decision makers in a collective action, excluding the elderly and children, is much less (Marlowe, 2005). These findings justify the assumption of group size $n=16$. However, group size variation among hunter-gatherer societies is large ($SD=38.28, Min=13, Max=250$; Marlowe, 2005). Therefore, we examine the sensitivity of other assumptions on group size $n$.

First, assuming a relatively small group size ($n=8$), $\alpha_{i}$ evolves to a very high value, with the rates of both prosocial behaviors close to 1 (Figure S13). In other words, when the population is composed of small subgroups, all members in a group will be highly socialized agents and realize a strong cooperative society. Of particular note is that cooperation evolves in most parameter spaces in the model with non-exapted $\alpha_{d}$ alone (Model 2, second left column). While the values of $\alpha_{i}$ are not much high, $\alpha_{d}$ evolves to large values in a neutral society with no injunctive norm pressure ${(v}_{x},v_{y})=(0.0, 0.0)$, with about half of the population cooperating in the last generation. This means that under small group sizes, conformity becomes adaptive. This will yield a moderately cooperative society that can be a path to a large-scale cooperative society sustained by injunctive norms.

Although there is no significant qualitative difference, larger group sizes lead to only more limited conditions under which cooperation evolves. For the genetic values of the simulations in which cooperation evolves, we can see that $\alpha_{d}$ is positively selected, not only $\alpha_{i}$. Maintaining cooperation in larger groups may require both the injunctive and descriptive norm-psychologies to work in a complementary manner. These findings are consistent with those of Andresguzman et al. (2007).

Figure S13: Summary result for the setting of **smaller group size (**$\boldsymbol{n=8}$**)**. Heatmap of $x$, $y$, $\alpha_{i}$ and $\alpha_{d}$ for different normative values $(v_{x}, v_{y})$, and 5 models. Other parameters: $m=0.5$. Shown are averages based on 10 runs for each parameter combination.


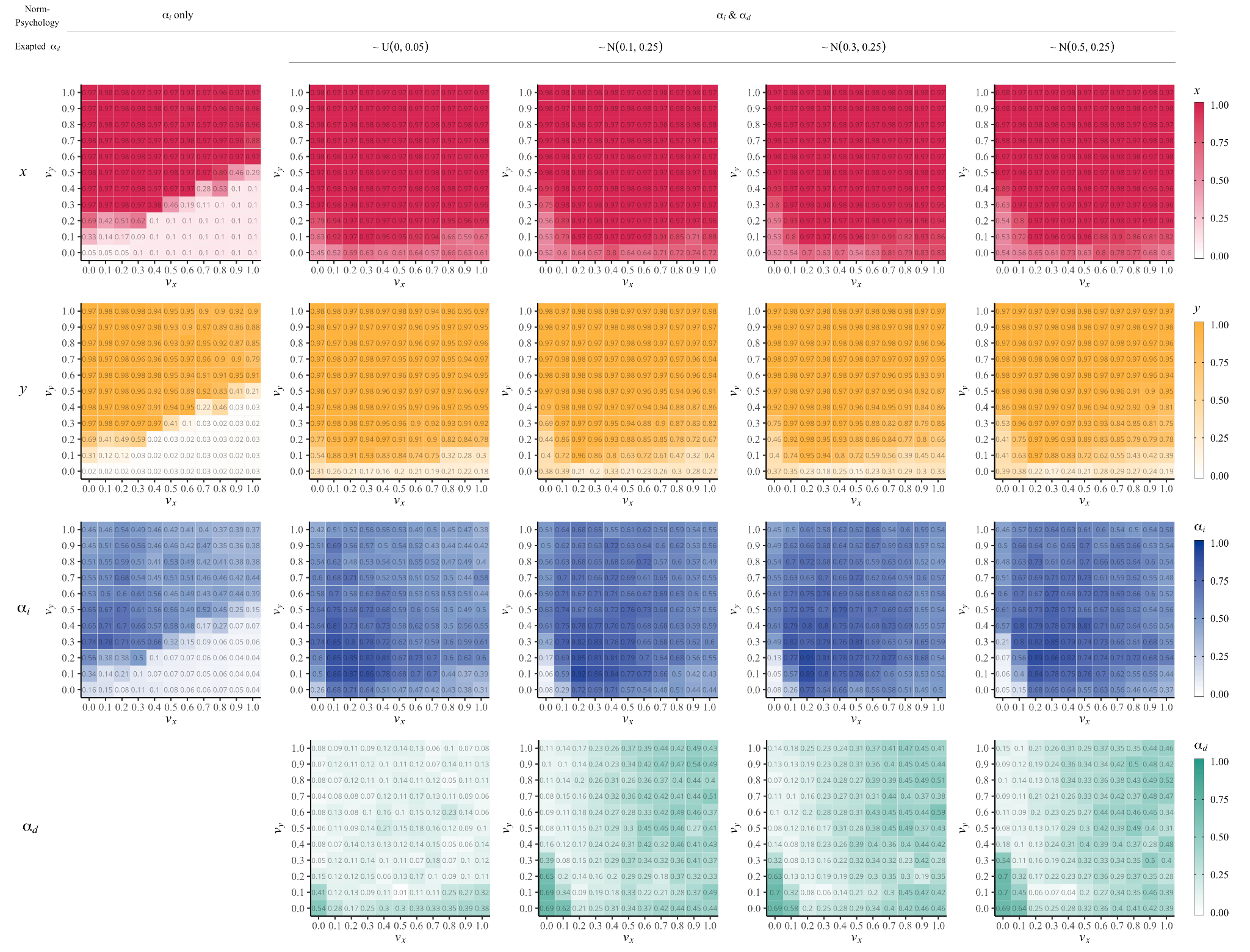


Figure S14: Summary result for the setting of **larger group size (**$\boldsymbol{n=24}$**)**. Heatmap of $x$, $y$, $\alpha_{i}$ and $\alpha_{d}$ for different normative values $(v_{x}, v_{y})$, and 5 models. Other parameters: $m=0.5$. Shown are averages based on 10 runs for each parameter combination.


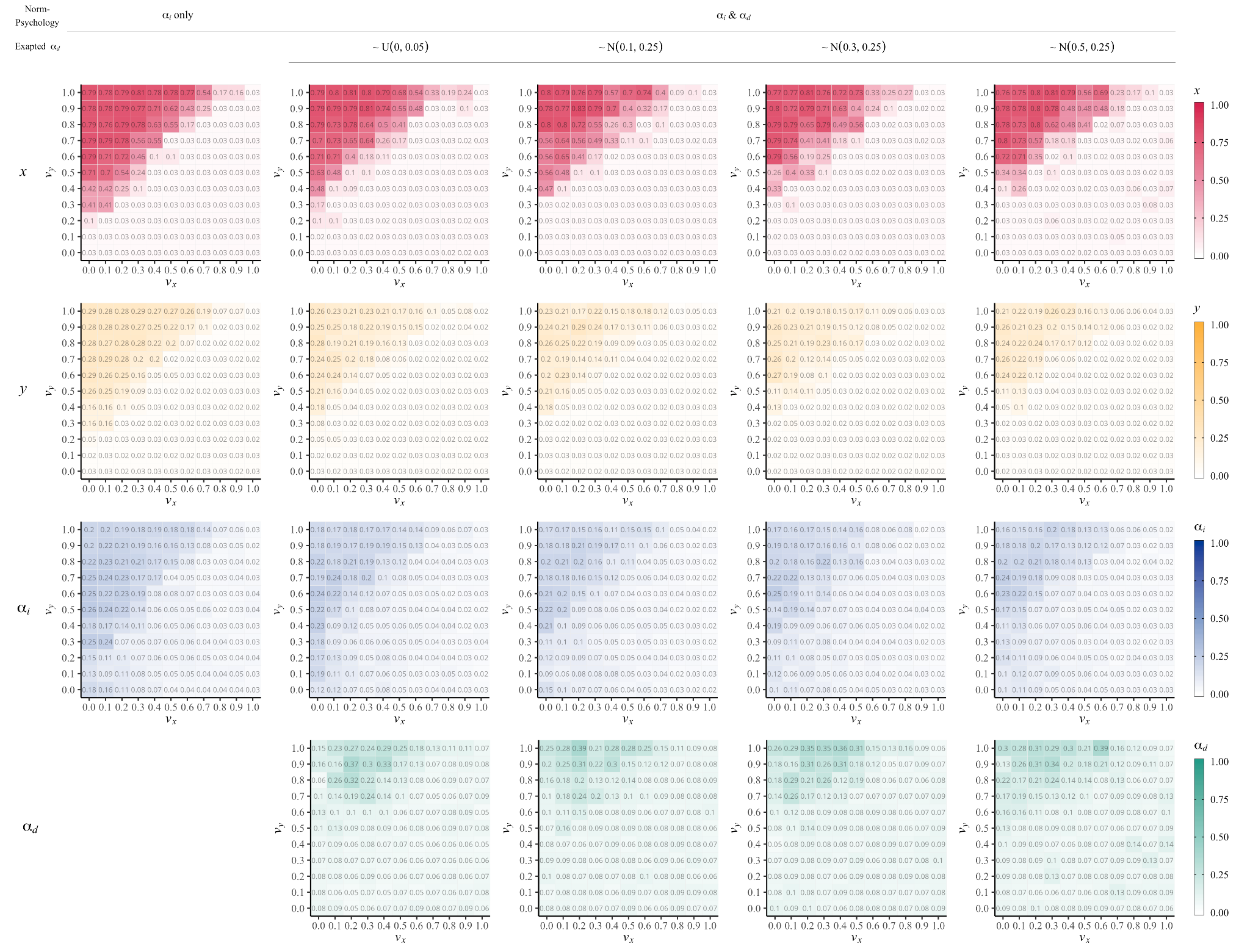


### Figure S15-16: Effect of migration rate $m$

In the main text, we present the results of an agent-based simulation with the group size parameter $m=0.5$. This setting can be taken to reflect the process of migration of women to other societies, which was typical in a patrilineal society of ancestral humans. Indeed, the foragers are highly mobile according to ethnographical observation (Marlowe, 2005): the median number of moves per year is 7 in warm-climate forager groups, which have the median number of group size 25.58. Thusly, humans have mixed with other groups in their lives. Theoretically, the increase in migration reduces not only genetic but also cultural differences among groups, thus impeding the evolution of cooperation. On the other hand, it has been suggested that conformist social learning may maintain large cultural differences even in a society with a large influx of immigrants (Henrich & Boyd, 1998). Therefore, $\alpha_{d}$ in our models is expected to serve as a buffer against the diminishing of diversity among cultures and drive cultural group selection.

Figure S15 illustrates that compared to the results (with $m=0.5$) in the main text, cooperation evolves under a wider range of conditions as people migrate less between groups. Under norms hardly encouraging punishment (bottom of parameter space $(v_{x},v_{y})$for each heatmap), the assumption of conformity increases the number of cooperators compared to the model with $\alpha_{i}$ alone (left column in Figure S15). Likewise, under norms hardly promoting cooperation (left side of parameter space $(v_{x},v_{y})$for each heatmap), as we assume exaptation of $\alpha_{d}$ (the more to the right in columns), the mechanism behind cooperation shifts from $\alpha_{i}$ to alpha $\alpha_{d}$ (the more to the right in columns, left side of the third row is less bluish, while the fourth row is more greenish).

Figure S16 illustrates that the condition for the evolution of cooperation is more stringent as migration is more frequent. However, as predicted, conformity mitigates the condition (the more to the right in columns, reddish region of the first row becomes larger). In particular, under the assumption of strong exaptation (i.e., initial values of $\alpha_{d}\sim N(0.5, 0.25))$, a cooperative society based on conformity is observed. However, when the injunctive norm encourages more cooperation than punishment, the equilibrium of defection is maintained. This reflects the fact that a cooperative group formed by “nice guys” who are willing to cooperate is easily invaded by defectors.

Figure S15: Summary result for the setting of **smaller migration rate (**$\boldsymbol{m=0.25}$**)**. Heatmap of $x$, $y$, $\alpha_{i}$ and $\alpha_{d}$ for different normative values $(v_{x}, v_{y})$and 5 models. Other parameters: $n=16$. Shown are averages based on 10 runs for each parameter combination.


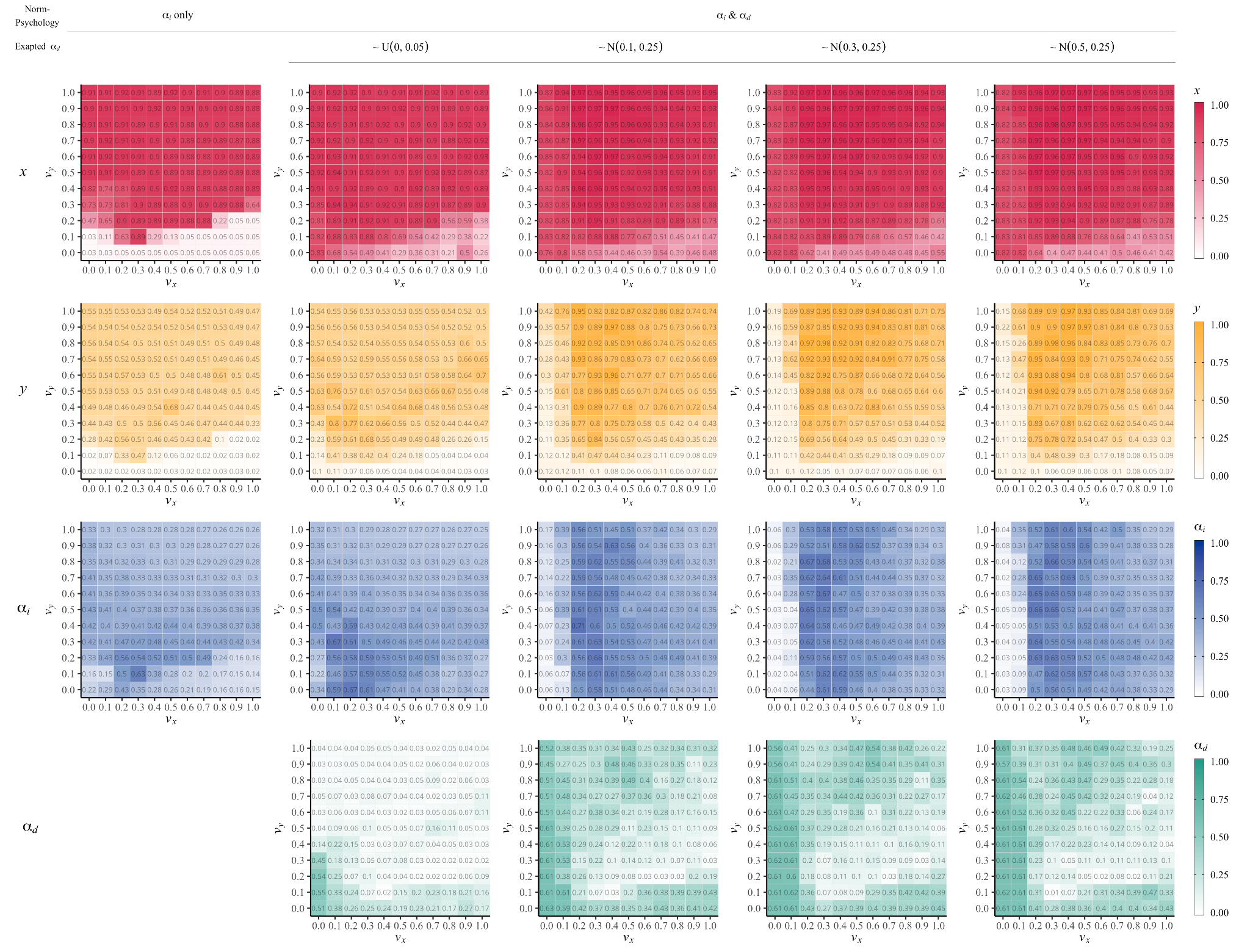


Figure S16: Summary result for the setting of **larger migration rate (**$\boldsymbol{m=0.75}$**)**. Heatmap of $x$, $y$, $\alpha_{i}$ and $\alpha_{d}$ for different normative values $(v_{x}, v_{y})$, and 5 models. Other parameters: $n=16$. Shown are averages based on 10 runs for each parameter combination.


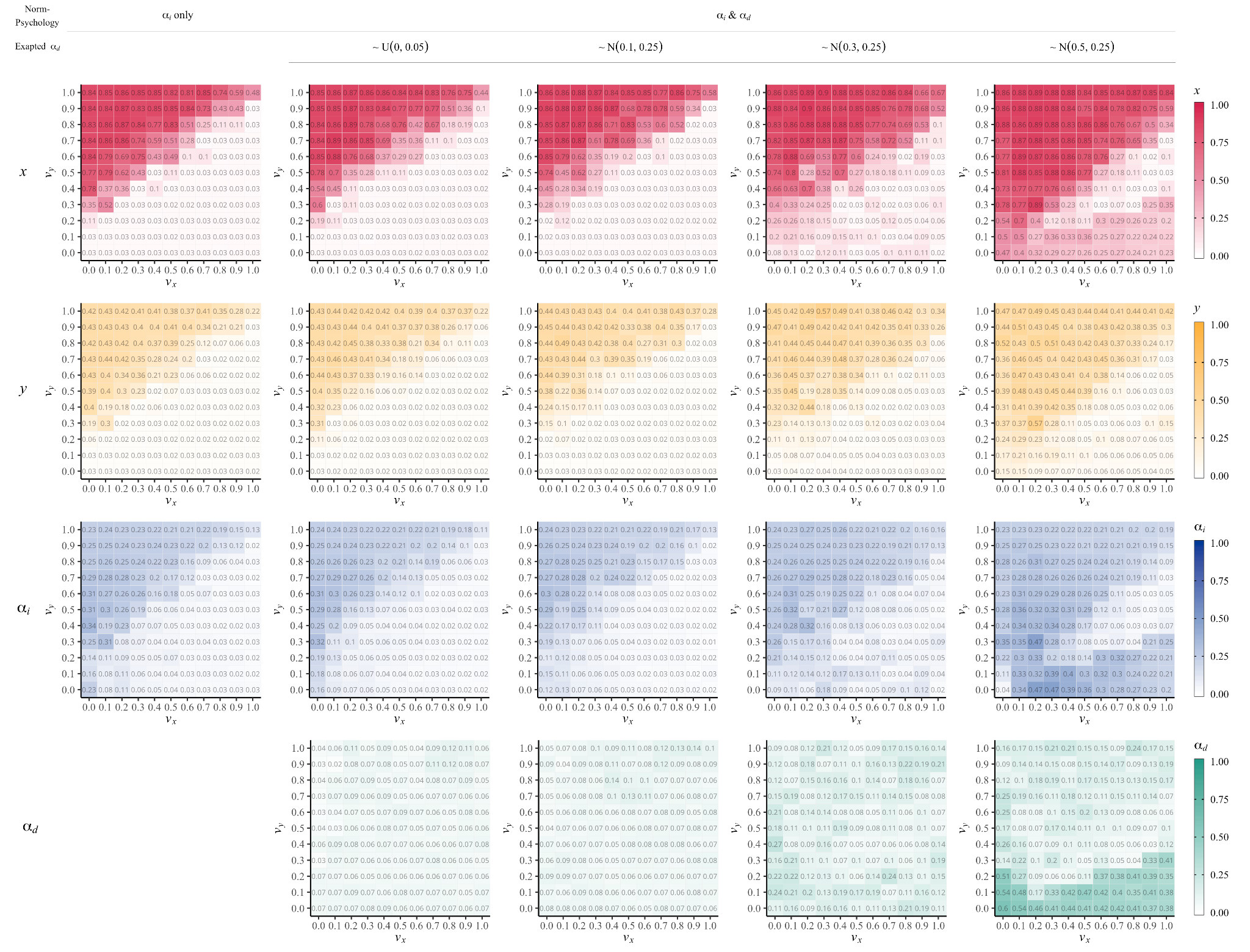


### **References**

Andresguzman, R., Rodriguezsickert, C., & Rowthorn, R. (2007). When in Rome, do as the Romans do: the coevolution of altruistic punishment, conformist learning, and cooperation. *Evolution and Human Behavior: Official Journal of the Human Behavior and Evolution Society*, *28*(2), 112–117.

Gavrilets, S., & Richerson, P. J. (2017). Collective action and the evolution of social norm internalization. *Proceedings of the National Academy of Sciences*, *114*(23), 6068–6073.

Henrich, J., & Boyd, R. (1998). The evolution of conformist transmission and the emergence of between-group differences. *Evolution and Human Behavior: Official Journal of the Human Behavior and Evolution Society*, *19*(4), 215–241.

Marlowe, F. W. (2005). Hunter-gatherers and human evolution. *Evolutionary Anthropology*, *14*(2), 54–67.
